# Mycorrhizal Fungi Influence on Mature Tree Growth: Stronger in High‐Nitrogen Soils for an EMF‐Associated Tree and in Low‐Nitrogen Soils for Two AMF‐Associated Trees

**DOI:** 10.1002/pei3.70055

**Published:** 2025-05-08

**Authors:** Inés Ibáñez, Morgan R. McPherson, Rima A. Upchurch, Donald R. Zak

**Affiliations:** ^1^ School for Environment and Sustainability University of Michigan Ann Arbor Michigan USA

**Keywords:** *Acer rubrum*, *Acer saccharum*, *arbuscular mycorrhizal fungi*, *ectomycorrhizal fungi*, *nitrogen*, *Quercus rubra*

## Abstract

The plant–mycorrhizal fungi relationship can range from mutualistic to parasitic as a function of the fungal taxa involved, plant ontogeny, as well as the availability of resources. Despite the implications this relationship may have on forest carbon cycling and storage, we know little about how mature trees may be impacted by mycorrhizae and how this impact may vary across the landscape. We collected growth data of two arbuscular mycorrhizal fungi (AMF)‐associated tree species, *Acer rubrum* and *A. saccharum* , and one ectomycorrhizal fungi (EMF)‐associated tree species, *Quercus rubra* , to assess how the mycorrhizal fungi–plant association may vary along a gradient of nitrogen (N) availability. Individual assessments of fungal taxa relative abundances showed non‐linear associations with tree growth; positive associations for the two AMF‐associated trees were mostly under low N, whereas positive to neutral associations for the EMF‐associated tree mainly took place at high N. Only *A. rubrum* exhibited greater tree growth with its tree soil‐specific mycorrhizal community when compared with predictions under a random mycorrhizal soil community. Because mycorrhizal fungi are likely to mediate how plants respond to warming, increasing levels of N deposition and of atmospheric CO_2_, understanding these relationships is critical to accurately forecasting tree growth.

## Graphical abstract

Because mycorrhizal fungi are likely to mediate how plants response to warming, increasing levels of N deposition and of atmospheric CO2, understanding these relationships is critical for generating accurate forecasts of tree growth under. Our results have quantified some of those relationships, information that can be used to reduce the uncertainty associated with predictions of future plant performance.

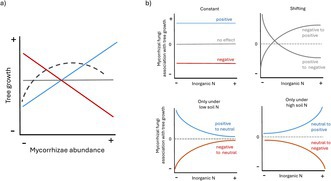

## 1 Introduction

Mycorrhizal fungi, specifically ectomycorrhizal (EMF) and arbuscular (AMF) mycorrhizal fungi, are essential to plants for nutrient and water acquisition, which increases photosynthetic activity and growth (Smith and Read 2010; Gavito et al. 2019). However, this association comes with costs; photosynthate is transferred from the plant to the fungi (Johnson et al. 1997; Karst et al. 2008), and in some cases, mycorrhizal fungi may even compete with the plant for soil nutrients (Alberton et al. 2007). Whether the outcome of this association is beneficial or not is highly context dependent, reflecting a dynamic balance between obtaining additional nutrients and losing photosynthate to the fungal symbiont (Allen et al. 2003; Bennett and Groten 2022). Despite its potential impact on carbon sequestration and nutrient cycling in forests (Treseder and Allen 2000; Terrer et al. 2016), this relationship has seldom been quantified under field conditions involving mature trees (~100 years old), which carry out most of the forest carbon uptake. Nevertheless, a comprehensive understanding and quantification of how this relationship takes place in late‐successional forests is crucial for predicting the impact of elevated atmospheric CO_2_, nitrogen deposition, and climate change on plant growth (Treseder 2004; Clemmensen et al. 2015). In this study, we examined the soil mycorrhizal fungi community associated with individual mature trees growing along a natural soil nitrogen (N) availability gradient. We then analyzed tree growth to determine the extent to which it is influenced by the dominant mycorrhizal taxa in their soils and how this relationship varies along a gradient of soil N availability.

**FIGURE 1:**
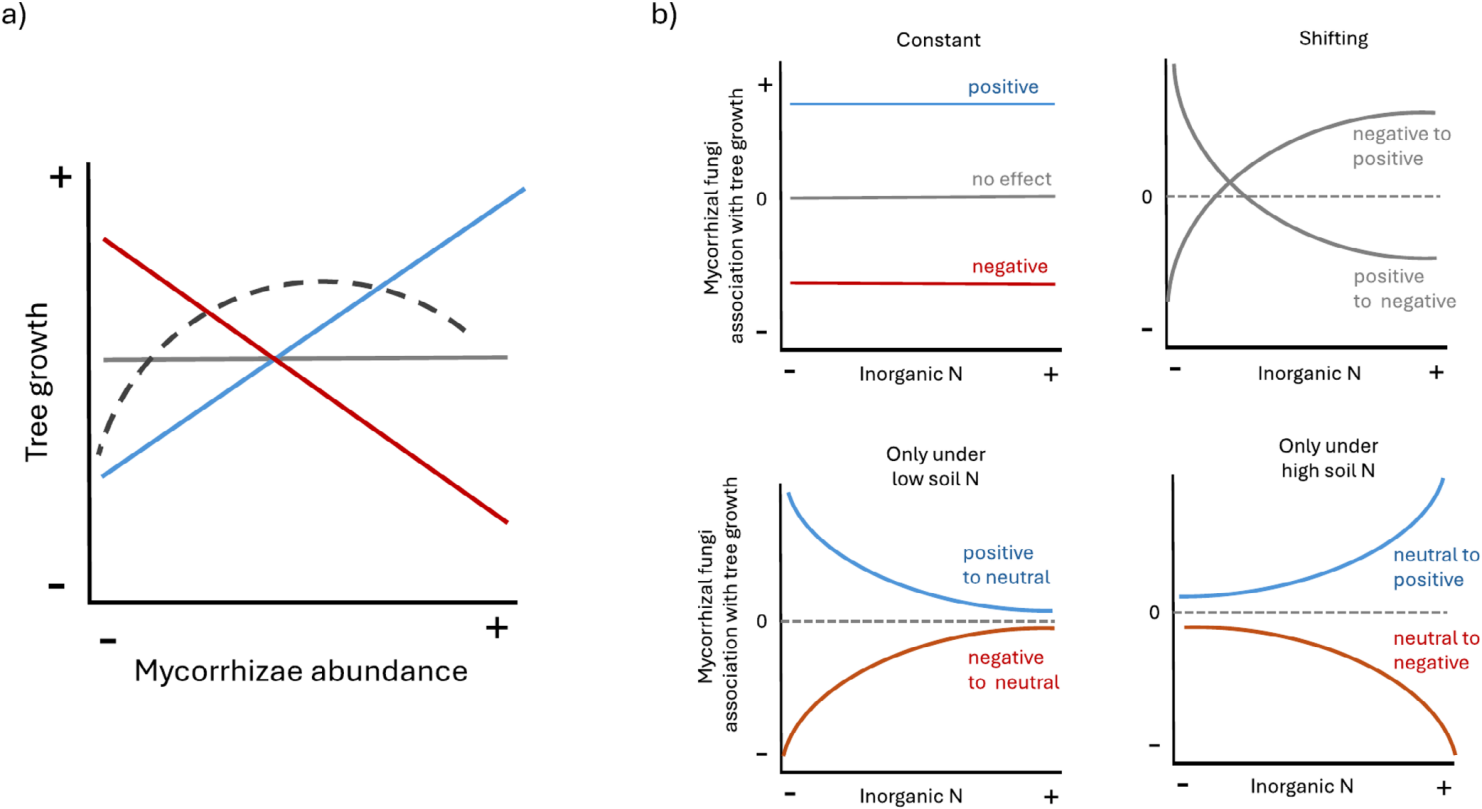
Potential associations between tree growth and relative mycorrhizal fungi abundance (a), and its potential changes along a gradient of nutrient availability (b).

Soil nutrient availability is a significant factor influencing the nature of the plant–mycorrhizal relationship (e.g., Jonsson et al. 2001; Cox et al. 2010; Bennett and Groten 2022). In N‐poor soil, if plants still have a photosynthate surplus (Bunn et al. 2024), additional access to N may be crucial for plant growth that would otherwise be limited by this nutrient. Conversely, in N‐rich soil, greater access to N via mycorrhizal fungi may be less critical to plant performance (Pena and Polle 2014; Allen et al. 2003). However, even in low‐N environments, if the photosynthate cost to the plant is too high, the association could ultimately be detrimental to plant growth (Kranabetter and MacKenzie 2010; Franklin et al. 2014; but see Bunn et al. 2024). Nutrient requirements for fungal hyphae are higher than those for plants (Allen et al. 2003); thus, in nutrient‐poor soils, nitrogen immobilization by mycorrhizal fungi might negatively affect plant performance (Lindahl et al. 2021). Moreover, in N‐rich soil, in which plant communities tend to experience more competition for light (Baribault and Kobe 2011), the N boost provided by mycorrhizal fungi could enhance a plant's competitive ability, especially when the photosynthate cost is less significant (Allen and Allen 1984; Hartnett et al. 1994). As a result, there are multiple alternatives wherein plants and mycorrhizae interact (Figure 1a), as well as how N availability might alter this interaction (Figure 1b).

These divergent associations have been attributed to the identity and diversity of the mycorrhizal species (Alberton et al. 2005; Hazard et al. 2017; Marro et al. 2022), which are likely to shift across gradients of soil N availability (e.g., McPherson et al. 2024; Pellitier et al. 2021). As a result, the composition of the mycorrhizal community will determine the nature of the association with the host plant (Pena and Tibbett 2024). Furthermore, the abundance of each taxon will determine the overall effect of the mycorrhizal community on the plant (Allen et al. 2003; Sim and Eom 2006).

Most research on tree species and mycorrhizal fungi has focused on seedlings and saplings (e.g., Teste et al. 2009; Hoeksema et al. 2010), whereas research on mature trees has largely been limited to biogeographic analyses (e.g., Anthony et al. 2022). The difficulty of conducting experiments with long‐lived plant species has hindered research on the impacts of this association in natural tree populations. Seedling studies have clearly documented how the relationship between trees and mycorrhizal fungi can shift from symbiotic to neutral, or even parasitic, as resources like N, phosphorus (P), and light vary (e.g., Koide 1991; Johnson et al. 2010; Ibáñez and McCarthy‐Neumann 2016). Experimental fieldwork with seedlings has also demonstrated similar shifts in response to changing resources (Zhou and Sharik 1997; Ibáñez and McCarthy‐Neumann 2014). However, seedlings and saplings have limited access to light and have not yet developed the extensive root systems that adult trees possess, and as a result, they might be more dependent, or vulnerable, to the effects of the mycorrhizal community (Booth and Hoeksema 2010).

Research involving mature trees has primarily been limited to biogeographic comparisons, for example correlating the abundance of specific EMF taxa with N availability (van der Linde et al. 2018) or examining tree performance in relation to the mycorrhizal taxa present at a given site (Anthony et al. 2022). As with seedlings, it is likely that the mycorrhizal‐plant relationship shifts as environmental conditions change, influencing how trees respond to N deposition (Cox et al. 2010; Morrison et al. 2016), benefit from elevated atmospheric CO_2_ (Alberton et al. 2007; Pellitier et al. 2021), or cope with climatic stress (Kipfer et al. 2012). A major untested assumption is that these associations have evolved to maximize a mutualistic outcome. However, these studies do not establish a clear connection between the specific mycorrhizal community colonizing an individual tree and that individual's performance (but see Birch et al. 2021).

In this study, we characterized the mycorrhizal community associated with three widely distributed tree species ( *Acer rubrum* , *A. saccharum* , and *Quercus rubra* ) in temperate forests of Eastern North America. We collected soil from individuals growing along a natural N availability gradient and analyzed tree growth as a function of the most abundant mycorrhizal taxa, as well as other biotic and abiotic factors that also affect tree performance. Our objective was to answer the following questions: (1) What is the relationship between specific mycorrhizal fungi and tree growth (Figure 1a)? (2) How is the mycorrhizal community, that is the abundance of the most dominant taxa, associated with tree growth? (3) Does the availability of soil N affect this relationship (Figure 1b)? And (4) Are the mycorrhizal communities under each tree associated with optimal tree growth? Answers to these questions and quantification of these relationships will shed new light on how plants and mycorrhizal fungi interact and how these interactions are shaped by soil nutrient availability, information critical to assess plant performance under current and future environmental conditions.

## 2 Materials and Methods

### 2.1 Nitrogen Gradient and Tree Species Sampled

To evaluate how soil N availability influences the mycorrhizal fungi–plant relationship (Figure 1b), we collected data across a natural gradient of soil inorganic nitrogen (N) availability, that is N mineralization, in the Manistee National Forest, Michigan, USA (Figure S1). The sampled trees are along a 75 km gradient of soil N availability. All samples were taken from even‐aged stands, 12 locations, that have regenerated after clear‐cutting in the early 20th century sharing similar sandy soil textures and climatic conditions (see Table S1). Differences in soil N availability arose due to physiographic variations influencing microclimate and nutrient retention (Zak et al. 1989). Across these locations, soil N ranges from 80 to 120 kg N^−1^ha^−1^, representing the full spectrum of N availability in this region (Zak and Pregitzer 1990).

We sampled individuals of three of the most abundant tree species: *Acer rubrum* (nine sampled locations), *A. saccharum* (six locations; we included two Acer species to ensure sampling across the whole N gradient), and *Q. rubra* (12 locations). *Acer* species form mycorrhizal associations with arbuscular mycorrhizal fungi (AMF), whereas *Quercus* predominantly associates with ectomycorrhizal fungi (EMF). These species are common and widespread in Eastern North American forests (see Table S2 for tree species ecological information). We also collected data on the neighborhood surrounding each tree; in a 10 m radius around each sampled tree, we identified and measured (diameter at breast height: dbh) all trees with dbh > 10 cm. Neighborhood data for each tree was summarized as conspecific or heterospecific basal area (BA cm^2^/m^2^) and as AMF‐associated trees BA or EMF‐associated trees BA.

### 2.2 Tree Rings Collection and Preparation

From mid‐June to mid‐July 2022, peak of the growing season in the region, we identified five canopy trees at each sampled location with a dbh larger than 10 cm. We extracted two cores, North and South sides, at dbh using Haglöf 5.15 mm increment borers (Haglöf Inc.; Madison, MS, USA). Cores were stored in paper straws until they could be air dried. Cores were then sanded with progressively finer sandpapers starting at 100 grit and ending at 1600 grit. We digitized the prepared samples using a flatbed scanner at a resolution of 1200 dpi. We measured annual ring width (growth) of digitized scans at a precision of 0.001 mm using the Cybis CooRecorder program. We then used Cybis Cdendro to crossdate samples and assemble different chronologies for each species. The expressed population signal (EPS; Wigley et al. 1984) for each species chronologies were 0.84 for *A. rubrum* (40 trees), 0.69 for *A. saccharum* (26 trees), and 0.89 for *Q. rubra* (60 trees). We then estimated historical dbh using ring width and the diameter of trees in 2022. To calculate yearly growth for our analyses, we computed the Basal Area Increment (BAI), for tree *i* and year *y*: ${\mathrm{BAI}}_{i,y}=\pi \left({\frac{{\mathrm{dbh}}_{i,y}}{4}}^{2}-{\frac{{\mathrm{dbh}}_{i,y-1}}{4}}^{2}\right).$ Links to data are found in the Supporting Information.

### 2.3 Soil Cores Collection and Processing

At the same time as tree sampling and after removing the Oi horizon, we collected eight 5‐cm‐deep soil cores in a 2‐m radius around each tree; cores were composited by individual tree. We passed soil through a 2‐mm sieve and immediately stored a subsample at −80°C for characterization of mycorrhizal communities. We used fresh subsamples to estimate inorganic N availability and air‐dried samples to measure soil P. We used 35‐day laboratory net N mineralization assays to re‐confirm soil inorganic N availability among our study sites (Vitousek et al. 1982; Zak et al. 1989). Specifically, we extracted inorganic N (NO_3_^−^ and NH_4_^+^) with 2 M KCl, then measured the initial and post‐incubation extracts using an AQ2 Discrete Analyzer (SEAL Analytical). Laboratory net N mineralization measurements are a robust representation of inorganic N availability because they are strongly correlated with in situ net N mineralization rates across these forest ecosystems (Zak et al. 1989; Zak and Pregitzer 1990). Soil pH was measured using a 1:1 ratio of air‐dried soil and deionized water, and C and N were determined using a CN analyzer (LECO) as previously described by (Argiroff et al. 2022). Soil phosphorus (P) was measured using the Bray‐Kurtz P1 method, using a Weak Bray extract. All soil P testing was conducted by A & L Great Lakes Laboratories (Fort Wayne, Indiana).

### 2.4 Mycorrhizal Community Characterization

We extracted genomic DNA from four 0.25 g subsamples of soil from around each tree using the PowerLyzer PowerSoil DNA Isolation Kit (Qiagen) with bead beading at 3000 rpm for 30 s and following the manufacturer's protocol. All extracted DNA quality and quantity were checked using gel electrophoresis and the Quant‐iT PicoGreen kit method (Thermo Fisher Scientific). For AMF, the 18S region was amplified using modified NS31 and AML2 primers, which are well characterized for AMF, to contain barcodes and Illumina dual‐indexed primers (Simon et al. 1992; Lee et al. 2008; Morgan and Egerton‐Warburton 2017). All PCRs were performed in triplicate following a modified protocol using Phusion High Fidelity DNA Polymerase and master mix (New England BioLabs, Argiroff et al. 2022; Taylor et al. 2016). Each PCR contained 5 μL High Fidelity Phusion 5 × buffer, 0.7 μL each primer (10 μM initial concentration), 2 μL dNTPs (20 mmol^−1^ initial concentration of each dNTP), 2 μL of template DNA (DNA concentration ranged from 8.5–55 ng/μL) and 0.2 μL of Phusion High Fidelity DNA Polymerase (2000 U/mL) brought to a final volume of 25 μL with 14.4 μL molecular‐grade water. PCR conditions consisted of an initial denaturation step at 95°C for 5 min, followed by 30 cycles of the following: 30 s at 95°C, 60 s at 69°C, and 45 s at 72°C, followed by a final extension step of 72°C for 3 min (McPherson et al. 2024). PCR libraries were sequenced with MiSeq 2 × 250 bp with v2 chemistry (Illumina) at the Advanced Genomics Core at the University of Michigan. For EMF, the ITS2 region was amplified using ITS4‐Fun and 5.8S‐Fun primers, which are well characterized for ITS (Argiroff et al. 2022; Taylor et al. 2016). All PCRs and subsequent processing were performed by the University of Michigan Microbiome Core.

For the AMF communities, we calculated amplicon sequence variants (ASVs; Callahan et al. 2017; Pauvert et al. 2019) using forward reads only as there was no overlap and previous studies have found that forward reads alone resolve AMF taxonomically (Davison et al. 2012; Morgan and Egerton‐Warburton 2017). ASVs were created using the ‘DADA2’ pipeline (Callahan et al. 2016; Rosen et al. 2012) with ‘cutadapt’ (Martin 2011) in R version 4.3.0 (R Core Team 2023). All reads were filtered and trimmed using the following parameters: manN = 0, truncLen = 240, maxEE = 1.75, trunQ = 2, minLen = 200. We then assigned taxonomy using a local blast environment with a modified MaarjAM database (Öpik et al. 2010). The modified MaarjAM (non‐type) database was edited to remove sequences with excessively short or long length and with any ambiguous bases. Taxonomic selections of virtual taxa (VTX; the taxonomic unit from the MaarjAM database) were assigned and filtered using a bitscore of 300 or higher as small portions of the 18S region had a high percent identity but with only partial overlap, resulting in an incorrect assignment by percent ID or e‐value alone; this step also removed suspected non‐AMF reads. We identified 147 ASVs and 24 VTXs between both maple species. For EMF communities, we followed the same protocol as with AMF, and calculated ASVs using forward reads. The processing pipeline used the following parameters for ITS: manN = 0, truncLen = 220, maxEE = 2, trunQ = 2, minLen = 200. We assigned taxonomy using ‘DADA2’ and the Unite database, and subset EMF from the entire ITS2 dataset for further analyses (Nilsson et al. 2019).

To evaluate how AMF and EMF communities changed across the net N mineralization gradient, we calculated Hellinger‐transformed relative abundances (Legendre and Gallager 2001) of AMF VTX and EMF genera by each sample using the “phyloseq” package in R (McMurdie and Holmes 2013). Guided by ordination analyses (using weights for the first three principal components which explained most of the variance), we selected the seven most abundant taxa across the gradient (VTX for *Acer* AMF communities, and genus for *Q. rubra* EMF communities). On average, those seven taxa abundances constituted more than half of the fungal community found under each tree, *A. rubrum* 83% (SD 13%), *A. saccharum* 54% (17%), and *Q. rubra* 63% (12%). We assumed that these are the most functionally relevant taxa, both providing nutrients and/or obtaining carbon from the plant (Allen et al. 2003). Since fungal communities are spatially stable over time even if they turnover across the seasons (Averill et al. 2019), we also assumed these are good representations of the mycorrhizal community in the soil at peak plant productivity.

### 2.5 Analysis Tree Growth

We analyzed tree growth, BAI, for the past four decades,1981–2021, as a function of the mycorrhizal community found in the soil under each individual tree. We also included well‐known predictors of tree growth in this region (Wang and Ibáñez 2022; Ibáñez et al. 2018), that is size (*ln*[*dbh*]), age, growth the previous year (lag effect using standardized *BAIS*_*i,t‐1*_), minimum temperature in May (*minMayT*), and net N mineralization (*Nmin*), the most limiting nutrient in this region (Zak et al. 1989; since these are relatively young soils, ~8000 years). Because the nature of the neighborhood surrounding a tree may also affect its performance (Hubert and Gehring 2008; Ibáñez and Rodríguez 2020), we included conspecific and heterospecific BA or AMF and EMF neighborhood BA as predictors. We then incorporated the abundance of the seven mycorrhizal taxa associated with each tree (Myco_1:7_) in linear and quadratic forms to assess linear or optimal relationships (Figure 1a; Gange and Ayres 1999). Finally, to assess any changes in this association along the N mineralization gradient (Figure 1b) we added an interaction term: *Nmin*•*Myco*. We tried several combinations of covariates and functions, such as grouping mycorrhizal taxa based on their peroxidase activity or morphotype. Below, we described the model with the best fit for *Q. rubra* based on deviance information criterion (DIC, Spiegelhalter et al. 2002, see Table S3). Best fit model for *A. saccharum* was similar, and the best fit model for *A. rubrum* included AMF and EMF neighbor trees BA. We also explored residuals to evaluate the addition of soil P as a covariate or of a spatially explicit random effect that would improve the fit, but we did not find any patterns. For each species independently, we modeled *BAI* for tree *i* in year *y* (*BAI*_*i,y*_) with a log‐normal likelihood:

(1) $${\mathrm{BAI}}_{i,y}\sim \text{Lnormal}\left(D_{i,y},{\sigma^{2}}_{i,y}\right)$$

And process model:

(2) $$\begin{array}{c} D_{i,y}=\alpha_{0}+\alpha_{1}\ln\left({\mathrm{dbh}}_{i}\right)+\alpha_{2}\cdot {\text{Nmin}}_{i}+\alpha_{3}\cdot {\text{BAIS}}_{i,y-1}+\alpha_{4}\cdot {\mathrm{age}}_{i,y}+\alpha_{4}\cdot {\text{minMayT}}_{y}\\ +\beta_{1:7}\cdot {\text{Myco}}_{i,1:7}+\gamma_{1:7}\cdot {\text{Myco}}_{i,1:7}^{2}+\mu_{1:7}\cdot {\text{Nmin}}_{i}\cdot {\text{Myco}}_{i,1:7} \end{array}$$

To account for an increase in variance as growth increases with a greater dbh, we estimated the variance (${\sigma^{2}}_{i,y}$) as a function of dbh: ${\sigma^{2}}_{i,y}=a+b∙\ln\left({\mathrm{dbh}}_{i,y}\right).$ Using a Bayesian approach all parameters were estimated from non‐informative prior distributions, $a\sim \text{logNormal}\left(1,1000\right),$ $\alpha_{*},\mu_{*},b\sim \text{Normal}\left(0,1000\right).$ To assess any codependences across mycorrhizal fungi taxa, coefficients associated with each taxa were estimated from uninformative multivariate normal distributions, $\beta_{1:7},\gamma_{1:7}\sim \text{MultiNormal}\left(0_{1:7},R_{7,7}\right)$ with variance–covariance matrix *R* ~ Wishart(I_7,7_,7).

The analyses were conducted using JAGS (Plummer 2021) and the rjags package in R (R Core Team 2023). Links to data used and code for the analyses are found in the Supporting Information. After a burn‐in period, 10,000 iterations, we ran three MCMC chains for 50,000 iterations until convergence was reached. The posterior parameter means, standard deviations, and 95% credible intervals were estimated across 50,000 additional iterations. We then used analyses estimates, coefficients' means, variances, and covariances to run simulations of tree performance under different scenarios.

### 2.6 Visualizing Results and Addressing Research Questions

To visualize our results, we ran a series of simulations using parameters from the analyses and range of values found in the data. To better assess the effect of the fungal community, all simulations were run for average values of the covariates, that is dbh, age, previous year standardized BAI, average May minimum temperature, and neighborhood BA (this last covariate only in *A. rubrum* simulations). Values used in the simulations can be found in Table S4. We ran four sets of simulations:

1. To visualize the relationship of each mycorrhizal taxon with tree growth, we estimated BAI along the range of mycorrhizal abundance for each mycorrhizal taxon, keeping N mineralization and the other six fungi at their average values.
2. To assess the relationship of the whole mycorrhizal community with tree growth, we estimated BAI at low and high values of each fungal taxon. Values were based on the average of the five highest and the five lowest abundances of each target mycorrhizae across our sampled trees. For N mineralization and the other 6 taxa, we calculated average values across those five trees, representing realistic levels of soil N and combinations of mycorrhizal abundances.
3. To assess how the relationship of different mycorrhizal communities with tree growth may change along a N mineralization gradient, using the mycorrhizal communities described in 2 we estimated tree growth along the N mineralization gradient and then calculated differences in predicted tree growth between high and low values of each mycorrhizal taxon across the N mineralization gradient.
4. To assess if the mycorrhizal community found at each site is optimizing tree growth, we first estimated BAI along the gradient of N mineralization values represented in the data using average abundance values of each mycorrhizal taxon, that is a random community, and compared those with estimated tree growth using values of the mycorrhizal community found at each location.

## 3 Results

After eliminating damaged tree cores and unsuccessful molecular analyses, we obtained 38 *A. rubrum* , 26 *A. saccharum* , and 57 *Q. rubra* matched tree and soil samples. Summaries of data, dbh, age, N mineralization, and mycorrhizal abundance can be found in Table S4. Model goodness of fit (*R* ^2^) varied from 0.74 for *A. rubrum* , 0.76 for *A. saccharum* , to 0.85 for *Q. rubra* (Figure S2). Including the mycorrhizal community as a predictor improved the goodness of fit by 11.4% for *A. rubrum* , 4.6% for *A. saccharum* , and 4.8% for *Q. rubra* . Parameter values from the analyses can be found in Table S5. Statistical significance was assessed on the basis of 95% credible intervals not overlapping with zero. Exploration of residuals did not show any spatial patterns nor associations with soil P (correlations in Figure S2).

**FIGURE 2:**
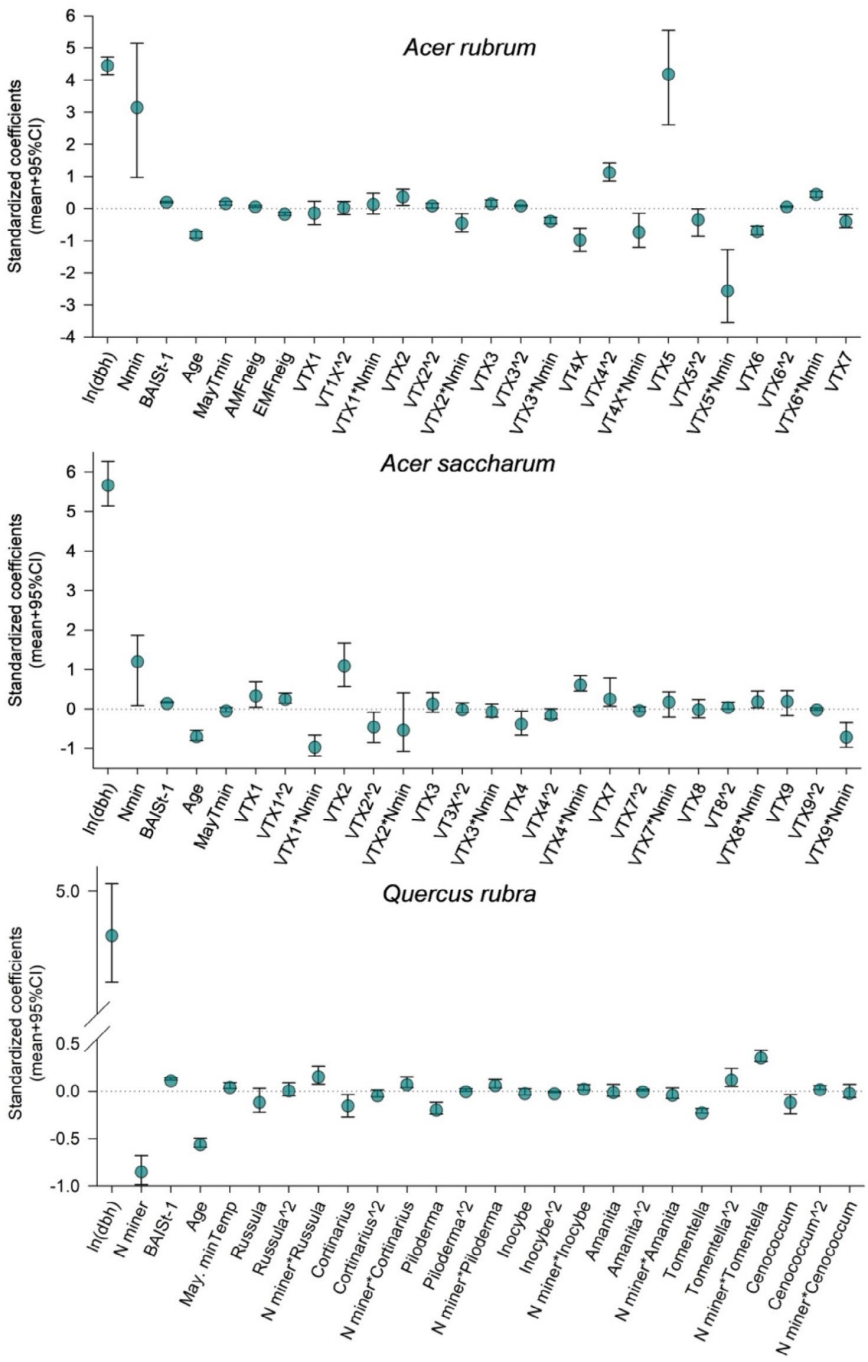
Standardized coefficient estimates for all predictors included in the analysis of tree growth along the N mineralization gradient. dbh: Tree diameter at breast height; Nmin: Nitrogen mineralization; BAIS_t‐1_: Previous year standardized growth; Age: Tree age; MayTmin: Average May minimum temperature; AMFneig or EMFneig: Basal area of tree neighbors in a 10 m radius that associate with AMF or with EMF.

For the three tree species, the strongest predictor of tree growth was size, *ln*(*dbh*) a positive association, whereas increasing age was associated with lower growth (Figure 2). The association between BAI and N mineralization, *Nmin*, was positive for the two *Acer* species and negative for *Quercus*, but note that the interaction terms between N mineralization and mycorrhizal abundance, *Nmin*•*Myco taxa*, were mostly positive (Figure 2). Previous year growth, *BAIS*_*y‐1*_, was also positively associated with current year growth in the three species. The association with minimum May temperature was positive for *A. rubrum* and *Q. rubra* . The associations between tree growth and mycorrhizal abundance were, in general, of lower magnitude than the rest of the predictors, with exceptions for particular taxa (Figure 2). Significant correlations between mycorrhizae‐related parameter pairs were few; one in *Q. rubra* and two in *A. rubrum* (Table S6).

**FIGURE 3:**
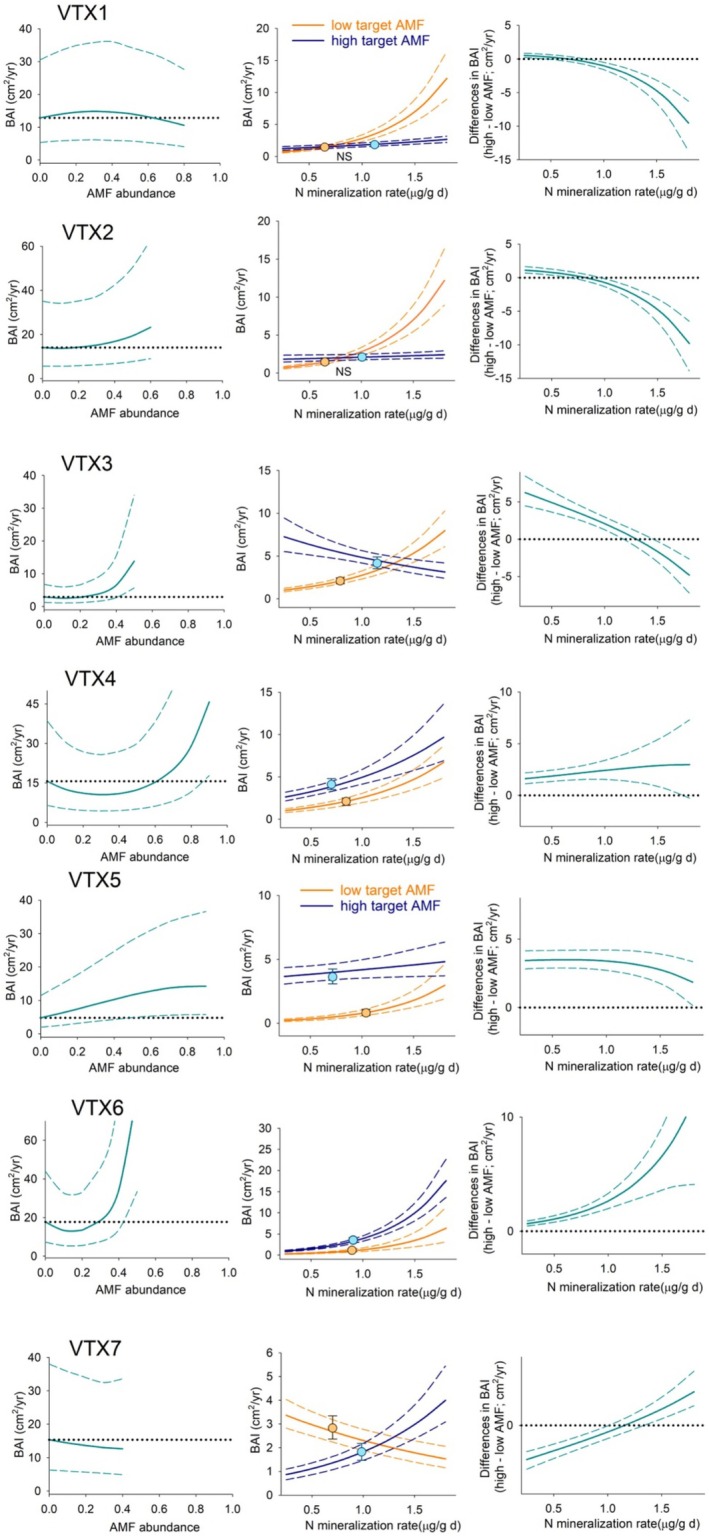
*Acer rubrum* growth, BAI, simulations (mean ± 95% PI). Left panels: Simulate BAI under the range of relative abundances of the target AMF while maintaining all other predictors, including the other six fungi, at average level. Central panels: Simulated BAI when growing with the mycorrhizal community, and N mineralization levels, associated with high and low values of the target AMF (symbols), and the same simulation along a N gradient (lines). NS: Not significant differences between symbols. Right panels: Differences from central panel lines.

**FIGURE 4:**
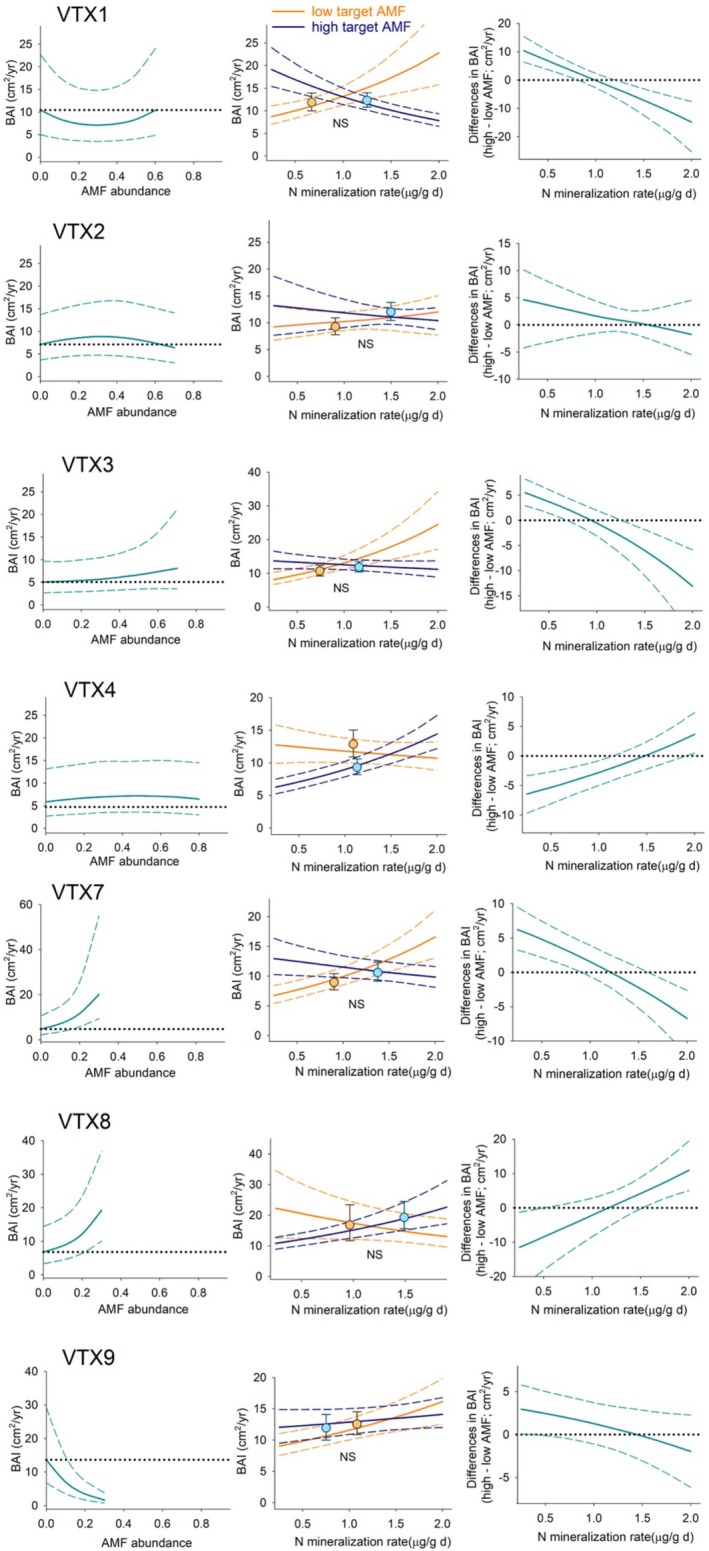
*Acer saccharum* growth, BAI, simulations (mean ± 95% PI). Left panels: Simulate BAI under the range of relative abundances of the target AMF while maintaining all other predictors, including the other six fungi, at average level. Central panels: Simulated BAI when growing with the mycorrhizal community, and N mineralization levels, associated with high and low values of the target AMF (symbols), and the same simulation along a N gradient (lines). NS: Not significant differences between symbols. Right panels: Differences from central panel lines.

**FIGURE 5:**
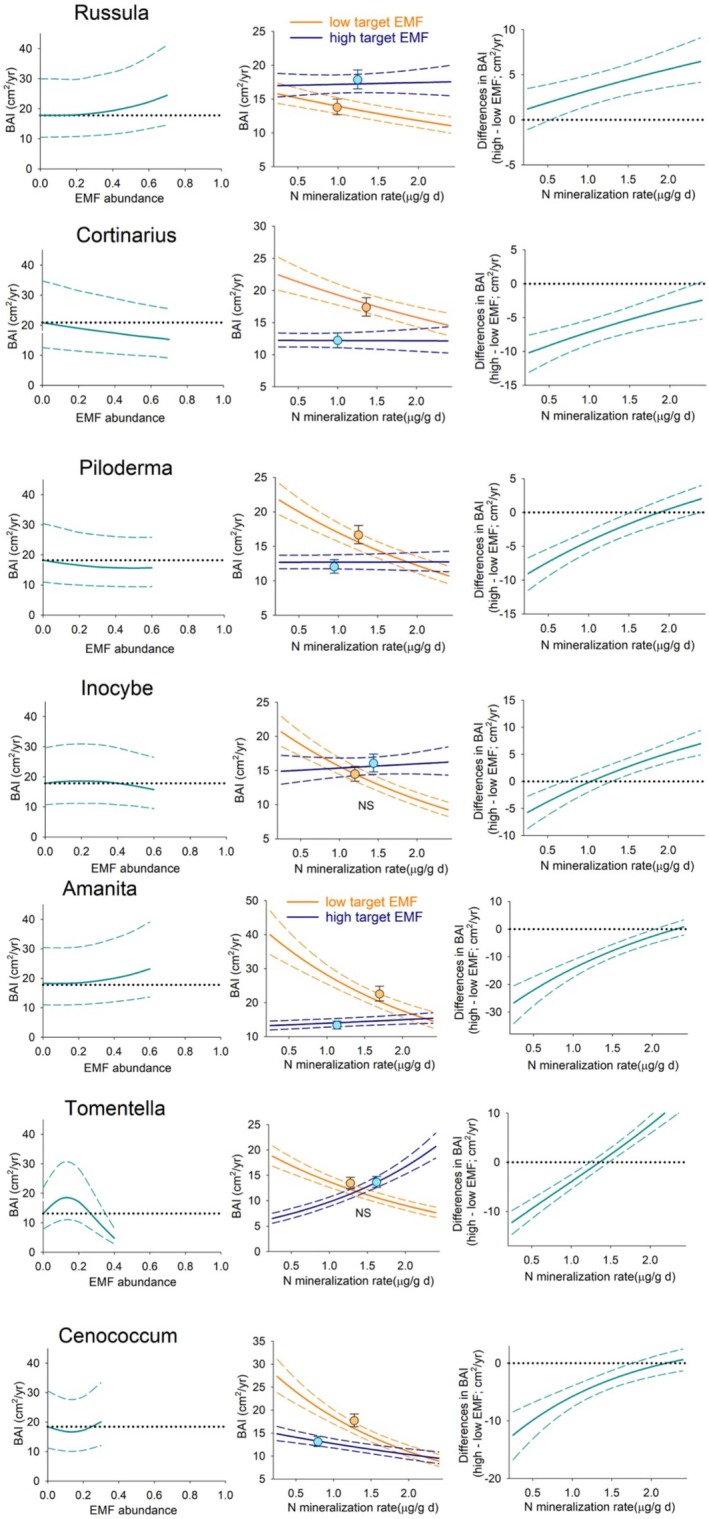
*Quercus rubra* growth, BAI, simulations (mean ± 95% PI). Left panels: Simulate BAI under the range of relative abundances of the target EMF while maintaining all other predictors, including the other six fungi, at average level. Central panels: Simulated BAI when growing with the mycorrhizal community, and N mineralization levels, associated with high and low values of the target EMF (symbols), and the same simulation along a N gradient (lines). NS: Not significant differences between symbols. Right panels: Differences from central panel lines.

When considering the specific associations between tree growth and mycorrhizal fungi, these varied across tree species and within each tree species across mycorrhizal taxa (Figure 2). To visualize those associations, we ran simulations of tree growth along the range of abundance values in the data and found mostly non‐linear associations (Figures 3, 4, 5 left panels). To have a more realistic assessment of these relationships in our next simulations, we estimated BAI under low and high levels of each taxon, keeping N mineralization and the other mycorrhizal taxa abundance within the range of values found under those conditions (Figures 3, 4, 5 central panels symbols). For *A. rubrum* , BAI estimates were higher under high abundance for four taxa (VTX3,4,5,6) and lower for one (VTX7), whereas in *A. saccharum*, there was only one instance when BAI estimates were significantly different, VTX4 with higher BAI under low abundance. Results for *Q. rubra* showed significant differences for five taxa, higher BAI under high abundance of *Russula* and higher BAI under low abundance for *Cortinarius*, *Piloderma*, *Amanita*, and *Cenococcum*.

We simulated the relationship between different mycorrhizal communities, low and high levels of each taxon, and tree growth along a gradient of N mineralization to assess how this relationship may shift as a function of nutrient availability (Figure 1b). Differences in tree growth between simulations at high and low abundance values of each mycorrhizal taxon (Figures 3, 4, 5 right panels) along the N gradient revealed a shift from positive to negative in three taxa for *A. rubrum* (VTX1,2,3) and *A. saccharum* (VTX1,3,7), while there was a switch from negative, or less positive to positive in two taxa (VTX6,7) for *A. rubrum* and two (VTX4,8) for *A. saccharum* . In *Q. rubra* , all taxa shifted to a positive, or more positive, association as N increased.

**FIGURE 6:**
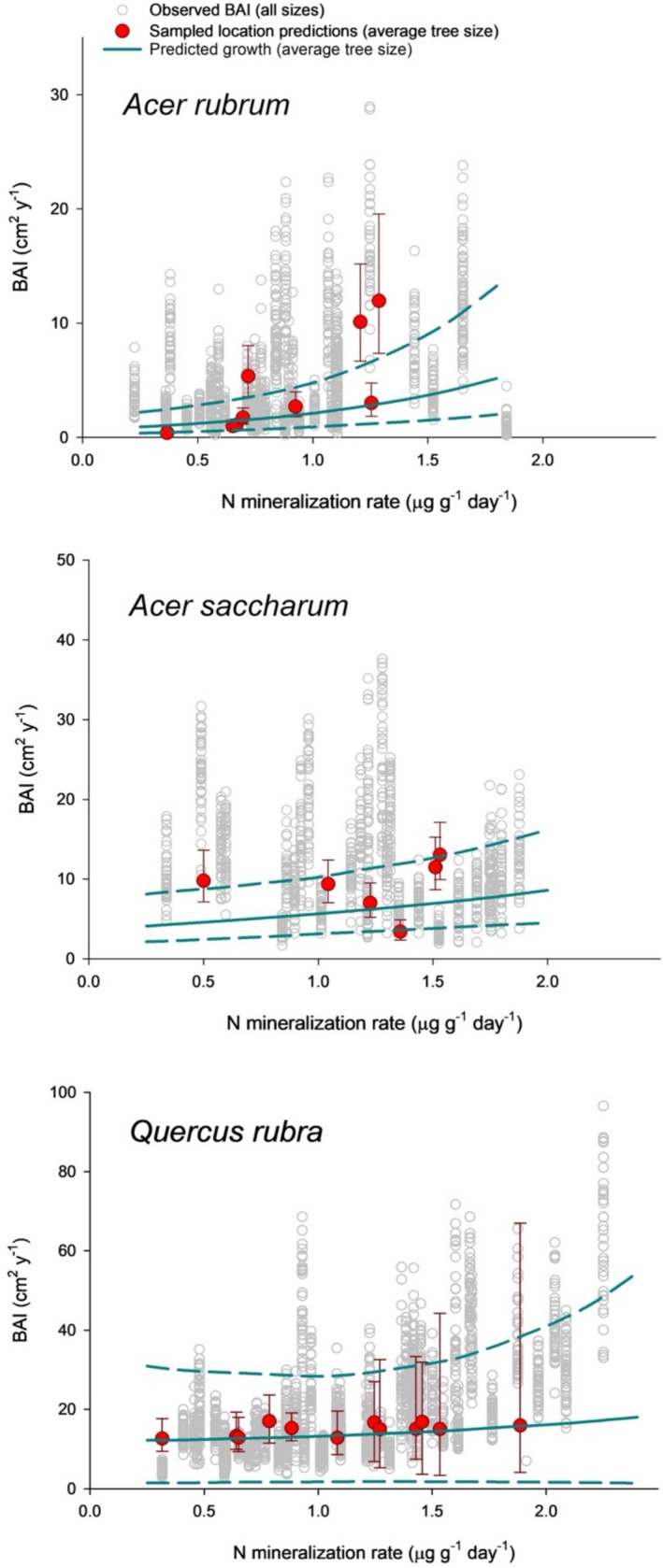
Tree growth along the N mineralization gradient sampled. Observed BAI across all trees and years (gray dots). Predicted growth across the N gradient of an average size and age tree with average relative mycorrhizal abundances (lines, mean‐solid, 95%PI‐dashed). Sampled location predictions (red symbols; mean ± 95% PI) of tree BAI under the average N mineralization and relative mycorrhizal abundance at that location.

To assess if the mycorrhizal fungi community we found under each tree optimized tree growth we looked at the differences in tree growth between being associated with a randomized mycorrhizal community (based on averages across all tree species; Figure 6 blue lines) and tree growth under the local mycorrhizal community (averaged across five sampled trees per site; Figure 6, red symbols). For *Acer rubrum* , predicted growth was consistently higher than the overall average, with red symbols falling above the prediction line. For *Acer saccharum* , there were no statistically significant differences, but in four of six locations, growth predictions were near the upper limit of the general trend. In contrast, for *Quercus rubra* , location‐specific predictions closely matched the overall mean, with red symbols aligning directly with the average prediction line.

## 4 Discussion

Mycorrhizal fungi are likely to mediate how plants respond to warming conditions (Kipfer et al. 2012), increasing levels of N deposition (Cox et al. 2010; Morrison et al. 2016) and atmospheric CO_2_ (Alberton et al. 2007; Pellitier et al. 2021). Therefore, understanding these associations is critical for generating accurate forecasts of tree performance as the Earth's climate changes (Tang et al. 2023). Our results have quantified some of those relationships and have led to new insights that can be used to reduce the uncertainty associated with predicting future tree growth. Our individual assessments of fungal abundance revealed various relationships with tree growth, mostly positive for *A. rubrum* , mostly neutral for *A. saccharum* , and mostly negative for *Q. rubra* . The abundance of these taxa also changed along the N availability gradient (McPherson et al. 2024; Pellitier et al. 2021), and so did their association with tree performance; there were more detrimental effects on growth under high N for the *Acer* species and more beneficial effects under high N for the EMF tree species. We also found that the impact of local communities did not differ from that of a random fungal community for two of the tree species, *A. saccharum* and *Q. rubra* . Only for *A. rubrum* were the location‐specific mycorrhizal communities associated with higher tree performance when compared with a random (average) community. Overall, our results demonstrate that the mycorrhizal fungi‐plant association, although modestly, also affects mature tree growth and that the nature and strength of these associations are mediated by the availability of soil N.

Mycorrhizal associations are considered essential for plants in acquiring soil nutrients and water, for conferring physical root protection, and for stimulating defense mechanisms to herbivores (e.g., Read and Perez‐Moreno 2003; Lehto and Zwiazek 2011; Vannette and Hunter 2013; but see Delavaux et al. 2017). These fungi extend the volume of soil accessible to plants at a much lower cost than expanding their root systems (Allen et al. 2003). In exchange, plants transfer energy in the form of photosynthate to the fungal symbiont. However, the nature of this association can vary along a spectrum that ranges from mutualistic to parasitic, depending on whether the additional nutrient uptake outweighs the photosynthate costs (Johnson et al. 1997; Ekblad et al. 2013). For mature trees, we found that this relationship is species‐specific (Figure 2) and that these associations are rarely linear, with both peaks and troughs of optimality (Figures 3, 4, 5 left panels). Models of AMF abundance and plant P uptake have revealed that non‐linear dynamics are possible (Gange and Ayres 1999) and a likely explanation for some of the contradictory results found in the literature (e.g., Francis and Read 1995; Bennett and Groten 2022). Furthermore, the beneficial effects of this association may be only apparent after certain levels of mycorrhizal abundance (Tonn and Ibáñez 2017; Suz et al. 2017). We also found a threshold dynamic in our analyses for *A. rubra* (VTX 3,4,5,6) and *A. saccharum* (VTX 7,8) wherein the benefits of the mycorrhizal associations were only significant at higher abundances. For one mycorrhizal taxon in *A. saccharum* (VTX9) and for one in *Q. rubra* (*Tomentella*), the negative effect also showed a threshold. Nevertheless, trees in natural settings are not exposed to a single mycorrhizal taxon, but to an entire community, and it is the combination of taxa that will affect plant growth (Sim and Eom 2006).

The impact of the entire mycorrhizal community has been mostly studied in tree seedlings (e.g., Koide and Dickie 2002; Albarracin et al. 2013). However, quantifying its effects in natural populations of mature trees has been elusive due to the difficulty of working with long‐life species as well as the challenge of isolating effects in a field setting (but see Birch et al. 2021). Overall, we found mycorrhizae explained a modest portion, 4 to 11%, of the variability in adult tree growth. Still, when all other factors affecting growth were maintained constant in the simulations, the impact of mycorrhizal communities associated with high and low abundances of each taxon resulted in divergent patterns across the three tree species (Figures 3, 4, 5 symbols in central panels). In *A. rubrum* , simulations revealed mostly higher growth at higher mycorrhizal fungi abundance; in *A. saccharum*, we found no differences between high and low mycorrhizal fungi abundance; and in *Q. rubra* , higher growth mostly took place at low mycorrhizal fungi abundance, which again may explain the diversity of results across studies e.g., (Smith and Read 2010 ; Lindahl et al. 2021). Higher BAI estimates were not always associated with the mycorrhizal community found at higher soil N. Furthermore, these associations changed along our natural N mineralization gradient (Figures 3, 4, 5 right panels), with mostly detrimental effects of higher mycorrhizal abundance at high N availability in the two AMF‐associated *Acer* species, indicating that AMF mycorrhizal fungi may be only beneficial under low soil nutrient conditions; in contrast, we found positive effects under high N availability in the EMF‐associated *Q. rubra* . If this effect on *Q. rubra* is due to fungal N immobilization at low soil N levels (Alberton et al. 2007; Näsholm et al. 2013) or to an increase in plant competitive advantage at high soil N availability (Weremijewicz et al. 2016) remains unresolved. Still, our findings reinforce previous experimental work with seedlings and non‐woody species indicating how the benefits of AMF and EMF took place at different levels of soil nutrient availability (e.g., Corrêa et al. 2011; Bunn et al. 2024); furthermore, our results quantify some of these associations, information that can be incorporated in forecasts of plant performance.

A major assumption of the plant–mycorrhizal symbiosis is that it has evolved to maximize a mutualistic outcome (Kiers and van der Heijden 2006; Kummel and Salant 2006). However, this optimization has rarely been tested, especially in mature trees like those in our study (Klironomos et al. 2011). Changes in the mycorrhizal community during succession suggest optimization between the host plant and fungal symbionts (Zangaro et al. 2003; Bachelot et al. 2018), and biogeographic differences in mycorrhizal communities linked to plant productivity indicate that optimization could be the case with adult trees (Anthony et al. 2022; Van Nuland et al. 2023). Nonetheless, this assumption has not been tested on mature trees when controlling for species and climatic differences, as we did in our study. Results from our simulations demonstrate that this maximization may not always take place. When we compared tree performance and location‐specific mycorrhizal fungi community with performance under an average community, we did not find differences in two of the three tree species (Figure 6). This lack of optimal symbiosis may be due to the generalist nature of the plant–mycorrhizal relationship (Davison et al. 2011; Rog et al. 2022) and of priority effects (Kennedy et al. 2009) that preclude plants from associating with the most optimal fungi.

Studying the mechanisms underlying the plant–mycorrhizal fungi relationship for mature trees growing in a natural setting is still unattainable, that is comparing mature trees with and without mycorrhizae. Nevertheless, we can leverage information collected from wild trees where the presence and abundance of mycorrhizal taxa found in their soils vary. Rather than only assessing the influence of a single taxon, our work also quantified the association between tree growth and the combination of mycorrhizal taxa coexisting in the soil around each tree. We cannot assume causation because we did not have “control” trees without mycorrhizal fungi, but we were still able to make inferences about how these fungal communities could impact plant performance. Our results revealed a diversity of associations and non‐linear dynamics, but in general, a stronger mutualistic association at high levels of soil N availability only for the EMF tree species, whereas the AMF tree species benefited more from mycorrhizae at low nutrient levels. These results are relevant in the context of predicting tree carbon uptake under varying environmental conditions, information needed to accurately predict plant performance under current and future climate conditions (Averill et al. 2014; Tedersoo et al. 2020). Taken together, our results are a first step in demonstrating that tree growth has a context‐dependent association with mycorrhizal fungi that is linked by the availability of soil N the nutrient that most limits forest growth across northern temperate forests. If other mature forests across this region exhibit a similar dynamic, mycorrhizal fungi are likely to mediate how these forests cycle and store carbon in response to warming, increasing levels of N deposition and atmospheric CO_2_, a response that will vary across the landscape as a function of soil N availability and the tree species involved.

## Conflicts of Interest

The authors declare no conflicts of interest.

## Supporting information


Data S1.
**Table S1.** Sampled locations soil data.
**Table S2.** Ecological information on the three tree species studied.
**Table S3.** Model selection for each of the three tree species analyzed based on deviance information criterion (DIC).
**Table S4.** Values used in the simulations, average across all the data or for each sampled location.
**Table S5.** Parameter values from the analyses, means, SDs, and 95% CIs.
**Table S6.** Correlations between parameters associated with mycorrhizal abundance.
**Figure S1.** Map with geographic information of sampled locations.
**Figure S2.** Models’ goodness of fit, predicted vs observed BAI. Correlations of residuals with soil P.

## Data Availability

Tree core data and analysis code can be found at the following link: https://doi.org/10.6073/pasta/f2cee6a41c56d7d66d490814d1f12e3e Molecular mycorrhizal data used in these analyses can be found at the following link: https://doi.org/10.5281/zenodo.13951756 Mycorrhizal sequences are deposited in the NCBI SRA BioProject (PRJNA714922) with the AMF SRA accession numbers SRR27482494–SRR27482565, and the EMF SRA accession numbers SRR33289102–SRR33289162.
