# Supplementary material for: Mycorrhizal Fungi Influence on Mature Tree Growth: Stronger in High‐Nitrogen Soils for an EMF‐Associated Tree and in Low‐Nitrogen Soils for Two AMF‐Associated Trees
Source: Plant Environ Interact. 2025 May 8;6(3):e70055. doi: 10.1002/pei3.70055 (PMC12059558; doi:10.1002/pei3.70055)
Supplement: Supplementary file 1 — Data S1. Table S1. Sampled locations soil data. Table S2. Ecological information on the three tree species studied. Table S3. Model selection for each of the three tree species analyzed based on deviance information criterion (DIC). Table S4. Values used in the simulations, average across all the data or for each sampled location. Table S5. Parameter values from the analyses, means, SDs, and 95% CIs. Table S6. Correlations between parameters associated with mycorrhizal abundance. Figure S1. Map with geographic information of sampled locations. Figure S2. Models’ goodness of fit, predicted vs observed BAI. Correlations of residuals with soil P. [file PEI3-6-e70055-s001.docx]

## Supporting Information

Article title: **Mycorrhizal fungi influence on mature tree growth: stronger in high-nitrogen soils for an EMF associated tree and in low-nitrogen soils for two AMF associated trees**

Authors: Inés Ibáñez, Morgan R. McPherson, Rima A. Upchurch, and Donald R. Zak

The following Supporting Information is available for this article:

**Data availability:** Links to data used in the analysis and model code. Mycorrhizal sequences are deposited in the NCBI SRA BioProject (PRJNA714922) with the AMF SRA accession numbers SRR27482494 - SRR27482565, and the EMF SRA accession numbers SRR33289102- SRR33289162.

Tree cores data and analysis code can be found at the following link:

<https://doi.org/10.6073/pasta/f2cee6a41c56d7d66d490814d1f12e3e>

Molecular mycorrhizal data used in these analyses can be found at the following link: <https://doi.org/10.5281/zenodo.13951756>

Mycorrhizal sequences are deposited in the NCBI SRA BioProject (PRJNA714922) with the AMF SRA accession numbers SRR27482494 - SRR27482565, and the EMF SRA accession numbers SRR33289102 - SRR33289162.

VTX ID numbers:

VTX1: VTX00084

VTX2: VTX00219

VTX3: VTX00166

VTX4: VTX00074

VTX5: VTX00113

VTX6: VTX00088

VTX7: VTX00385

VTX8: VTX00024

VTX9: VTX00115

The range of mycorrhizal fungi relative abundance range from 0 to 0.95 (*Acer rubrum*), 0 to 0.70 (*A. saccharum*), and 0-0.68 (*Quercus rubra*). Assisted by an ordination analysis we chose taxa with the highest relative abundances, these ranged between 0.35 and 0.95 (A. rubrum), 0.18 and 0.70 (*A. saccharum*), and 0.29 and 0.68 (*Q. rubra*), and taxa that were also present across the largest number of trees, 18 to 35 trees (*A. rubrum*), 5 to 26 trees (*A. saccharum*), and 38 to 60 trees (*Q. rubra*). The rest of the taxa had much lower relative abundances and were found in a smaller number of trees.

Supplementary Tables

**Table S1:** Sampled location level soil data. Geographic locations, latitude and longitude, and location averages for phosphorous (P in ppm), net N mineralization rate (*μ*g N g^-1^ d^-1^), soil pH, percentage of N, percentage of C, and percentage of soil moisture.

| Location ID | Avg. Latitude | Avg. Longitude | P | N min | soil pH | % N | % C | % soil moisture |
| --- | --- | --- | --- | --- | --- | --- | --- | --- |
| 3 | 44.26415582 | -86.17795378 | 12.3 | 0.70 | 3.69 | 0.16 | 4.00 | 9.6 |
| 6 | 44.2198641 | -85.66850176 | 14.9 | 0.47 | 5.46 | 0.15 | 2.07 | 18.6 |
| 7 | 44.19263836 | -85.67795255 | 11.4 | 0.86 | 4.25 | 0.15 | 2.70 | 9.3 |
| 9 | 44.32029192 | -85.97698 | 19.4 | 0.72 | 4.03 | 0.12 | 2.42 | 13.1 |
| 20 | 44.19483112 | -86.0913715 | 11.0 | 0.66 | 3.62 | 0.15 | 3.52 | 8.4 |
| 22 | 44.37322684 | -85.70831864 | 15.3 | 1.56 | 4.35 | 0.17 | 2.47 | 12.3 |
| 24 | 44.22227095 | -85.75187814 | 12.5 | 1.25 | 4.51 | 0.17 | 2.52 | 16.1 |
| 31 | 44.27203236 | -85.99245216 | 15.0 | 1.21 | 3.80 | 0.15 | 3.50 | 17.2 |
| 41 | 44.34688783 | -85.48180105 | 14.8 | 1.36 | 3.75 | 0.13 | 1.88 | 11.6 |
| 50 | 44.25659468 | -86.08361476 | 13.7 | 0.36 | 3.60 | 0.16 | 3.83 | 6.7 |
| 58 | 44.310452 | -85.8967945 | 11.8 | 0.94 | 3.67 | 0.12 | 2.33 | 15.4 |
| 100 | 44.34707284 | -85.4833218 | 13.7 | 1.47 | 4.39 | 0.16 | 2.14 | 14.7 |

**Table S2**: Ecological information on the three tree species studied, main mycorrhizal type associated with their roots, the range of dbhs and ages we sampled, and locations where that species was present along the gradient we surveyed.

| Tree Species | Shade tolerance | Growth | Myco. type | Range of dbhs | Range of ages | Locations present (Table S1) |
| --- | --- | --- | --- | --- | --- | --- |
| *Acer rubrum* | tolerant | moderately fast | AMF | 12-59 | 41-113 | 3,7,9,20,24,31,  41,50,58 |
| *Acer saccharum* | very tolerant | very slow | AMF | 27-74 | 58-104 | 6,7,22,24,41,  100 |
| *Quercus rubra* | intermediate | relatively fast | EMF | 21-140 | 34-110 | all |

| Tree Species | Range conspecific neighbors  (BA m^2^/ha) | Range heterospecific neighbors  (BA m^2^/ha) | Range AMF neighbors  (BA m^2^/ha) | Range EMF neighbors  (BA m^2^/ha) |  |  |
| --- | --- | --- | --- | --- | --- | --- |
| *Acer rubrum* | 0-30.42 | 0-95.03 | 0-30.42 | 0-67.28 |  |  |
| *Acer saccharum* | 0-31.05 | 0-68.56 | 0-31.05 | 0-67.28 |  |  |
| *Quercus rubra* | 0-70.52 | 0-72.9 | 0-59.57 | 0-70.52 |  |  |

**Table S3**: Model selection for each of the three tree species analyzed based on deviance information criterion (DIC). Selected model in bold.

| *Acer rubrum* | |
| --- | --- |
| Model | DIC |
| ln(dbh)+Nminer+BAISt-1+Age+Maytemp+conspecifiBA+HeterospecificBA | 6159 |
| ln(dbh)+Nminer+BAISt-1+Age+Maytemp+AMFNeighBA+EMFNeighBA | 6127 |
| AMF | 7442 |
| AMF + ln(dbh) | 6310 |
| AMF + ln(dbh) + Nmin | 6310 |
| AMF + ln(dbh) + Nmin + BAIS | 6035 |
| AMF + ln(dbh) + Nmin + BAIS + age | 5896 |
| AMF + ln(dbh) + Nmin + BAIS + age + Maytemp | 5883 |
| AMF + ln(dbh) + Nmin + BAIS + age + Maytemp + AMFNeighBA + EMFNeighBA | 5767 |
| AMF + ln(dbh) + Nmin + BAIS + age + Maytemp + AMFNeighBA + EMFNeighBA + Nmin*AMF | 5639 |
| AMF + ln(dbh) + Nmin + BAIS + age + Maytemp + AMFNeighBA + EMFNeighBA + Nmin*AMF + AMF^2 | **5425** |
|  |  |
| *Acer saccharum* | |
| Model | DIC |
| ln(dbh)+Nminer+BAISt-1+Age+Maytemp+conspecifiBA+HeterospecificBA | 5457 |
| ln(dbh)+Nminer+BAISt-1+Age+Maytemp+AMFNeighBA+EMFNeighBA | 5459 |
| AMF | 6301 |
| AMF + ln(dbh) | 5783 |
| AMF + ln(dbh) + Nmin | 5769 |
| AMF + ln(dbh) + Nmin + BAIS | 5548 |
| AMF + ln(dbh) + Nmin + BAIS + age | 5386 |
| AMF + ln(dbh) + Nmin + BAIS + age + Maytemp | 5385 |
| AMF + ln(dbh) + Nmin + BAIS + age + Maytemp + AMFNeighBA + EMFNeighBA | 5340 |
| AMF + ln(dbh) + Nmin + BAIS + age + Maytemp + AMFNeighBA + EMFNeighBA + Nmin*AMF | 5259 |
| AMF + ln(dbh) + Nmin + BAIS + age + Maytemp + AMFNeighBA + EMFNeighBA + Nmin*AMF + AMF^2 | 5212 |
| AMF + ln(dbh) + Nmin + BAIS + age + Maytemp + Nmin*AMF + AMF^2 | **5209** |
|  |  |
| *Quercus rubra* | |
| **Model** | **DIC** |
| EMF | 16344 |
| EMF by morphotype | 16468 |
| EMF + ln(dbh) | 14165 |
| EMF + ln(dbh) + Nminr | 14123 |
| EMF + ln(dbh) + Nminr + BAISt-1 | 13849 |
| EMF + ln(dbh) + Nminr + BAISt-1 + conspecificNeighborsBA | 13856 |
| EMF + ln(dbh) +Nminr + BAISt-1 + age | 13301 |
| EMF + ln(dbh) +Nminr + BAISt-1 + age + AMFneighborsBA | 13340 |
| EMF + ln(dbh) +Nminr + BAISt-1 + age + allneighborsBA | 13320 |
| EMF + ln(dbh) +Nminr + BAISt-1 + age + May tmep | 13256 |
| EMF + ln(dbh) +Nminr + BAISt-1 + age + May tmep + EMF*Nmin | 13185 |
| lln(dbh) +Nminr + BAISt-1 + age + May tmep | 13472 |
| **EMF + EMF^2 + ln(dbh)**  **+Nminr + BAISt-1 + age + May tmep + EMF*Nmin** | **12818** |

**Table S4:** Values used in the simulations, average across all the data or for each sampled location. Mycorrhizal abundance values were sampled from normal distributions based on these means and SD. These are overall and location level averages of the Hellinger-transformed relative abundances, i.e., they may add to more than one.

| Species | | | | | | dbh (cm) | | | | | | | Age (years) | | | | | | | BAIS | | | | | Average May minimum temperature (°C) | | | | | | | | | | | | | BAI  AMF  (cm^2^/m^2^) | | | | | | | | BAI  EMF  (cm^2^/m^2^) | | | | | | | |  | | | |
| --- | --- | --- | --- | --- | --- | --- | --- | --- | --- | --- | --- | --- | --- | --- | --- | --- | --- | --- | --- | --- | --- | --- | --- | --- | --- | --- | --- | --- | --- | --- | --- | --- | --- | --- | --- | --- | --- | --- | --- | --- | --- | --- | --- | --- | --- | --- | --- | --- | --- | --- | --- | --- | --- | --- | --- | --- | --- |
| *A.rubrum* | | | | | | 28 | | | | | | | 83 | | | | | | | 0 | | | | | 5.23 | | | | | | | | | | | | | 6.2 | | | | | | | | 18.55 | | | | | | | |  |  |  |  |
| *A.saccharum* | | | | | | 45 | | | | | | | 82 | | | | | | | 0 | | | | | 5.23 | | | | | | | | | | | | | - | | | | | | | | - | | | | | | | |  |  |  |  |
| *Q. rubra* | | | | | | 52 | | | | | | | 74 | | | | | | | 0 | | | | | 5.23 | | | | | | | | | | | | | - | | | | | | | | - | | | | | | | |  |  |  |  |
| Average (and SD) abundance (proportion) of the Mycorrhizal Data (across all locations) | | | | | | | | | | | | | | | | | | | | | | | | | | | | | | | | | | | | | | | | | | | | | | | | | | | | | | | | | |
|  | | | | | | VTX1 | | | | | | | | | VTX2 | | | | | | | | | VTX3 | | | | | | | VTX4 | | | | | | | | VTX5 | | | | | | | | | VTX6 | | | | | | | VTX7 | | |
| *A.rubrum* | | | | | | 0.36 (0.16) | | | | | | | | | 0.21 (0.19) | | | | | | | | | 0.08 (0.14) | | | | | | | 0.42 (0.20) | | | | | | | | 0.59 (0.25) | | | | | | | | | 0.079  (0.10) | | | | | | | 0.089 (0.07) | | |
|  | | | | | | | | | | | | | | | | | | | | | | | | | | | | | | | | | | | | | | | | | | | | | | | | | | | | | | | | | |
|  | | | | | | VTX1 | | | | | | | | | VTX2 | | | | | | | | | VTX3 | | | | | | | VTX4 | | | | | | | | VTX7 | | | | | | | | | VTX8 | | | | | | | VTX9 | | |
| *A.saccharum* | | | | | | 0.27 (0.14) | | | | | | | | | 0.42 (0.13) | | | | | | | | | 0.29 (0.20) | | | | | | | 0.35  (0.19) | | | | | | | | 0.13  (0.07) | | | | | | | | | 0.08 (0.03) | | | | | | | 0.11 (0.04) | | |
|  | | | | | | | | | | | | | | | | | | | | | | | | | | | | | | | | | | | | | | | | | | | | | | | | | | | | | | | | | |
|  | | Russula | | | | | | | | | Cortinarius | | | | | | | | | | | Piloderma | | | | | | | | Inocybe | | | | | | | Amanita | | | | | | | Tomentella | | | | | | | | Cenococcum | | | | | |
| *Q. rubra* | | 0.32 (0.14) | | | | | | | | | 0.25 (0.18) | | | | | | | | | | | 0.18 (0.22) | | | | | | | | 0.08  (0.13) | | | | | | | 0.1 (0.1) | | | | | | | 0.11  (0.07) | | | | | | | | 0.05  (0.08) | | | | | |
| Sampled location level N mineralization (μg/g d ) averages and location average abundance (Hellinger-transformed relative abundances) of the Mycorrhizal Data | | | | | | | | | | | | | | | | | | | | | | | | | | | | | | | | | | | | | | | | | | | | | | | | | | | | | | | | | |
| *A.rubrum* | | | N | | | | | | | | | VTX1 | | | | | | | | | VTX2 | | | | | | | VTX3 | | | | | | | VTX4 | | | | | | | VTX5 | | | | | | | | VTX6 | | | | VTX7 | | | |
| Site 3 | | | 0.69 | | | | | | | | | 0.38 | | | | | | | | | 0.08 | | | | | | | 0.07 | | | | | | | 0.22 | | | | | | | 0.79 | | | | | | | | 0.07 | | | | 0.008 | | | |
| Site 7 | | | 0.65 | | | | | | | | | 0.44 | | | | | | | | | 0.31 | | | | | | | 0.05 | | | | | | | 0.43 | | | | | | | 0.44 | | | | | | | | 0.01 | | | | 0.1 | | | |
| Site 9 | | | 0.71 | | | | | | | | | 0.42 | | | | | | | | | 0.24 | | | | | | | 0.03 | | | | | | | 0.62 | | | | | | | 0.46 | | | | | | | | 0.05 | | | | 0.05 | | | |
| Site 20 | | | 0.66 | | | | | | | | | 0.3 | | | | | | | | | 0.02 | | | | | | | 0.006 | | | | | | | 0.4 | | | | | | | 0.78 | | | | | | | | 0.14 | | | | 0.07 | | | |
| Site 24 | | | 1.01 | | | | | | | | | 0.39 | | | | | | | | | 0.45 | | | | | | | 0.44 | | | | | | | 0.02 | | | | | | | 0.12 | | | | | | | | 0.03 | | | | 0.23 | | | |
| Site 31 | | | 1.2 | | | | | | | | | 0.31 | | | | | | | | | 0.2 | | | | | | | 0.12 | | | | | | | 0.46 | | | | | | | 0.67 | | | | | | | | 0.1 | | | | 0.11 | | | |
| Site 41 | | | 1.25 | | | | | | | | | 0.48 | | | | | | | | | 0.42 | | | | | | | 0.11 | | | | | | | 0.47 | | | | | | | 0.41 | | | | | | | | 0.006 | | | | 0.1 | | | |
| Site 50 | | | 0.36 | | | | | | | | | 0.09 | | | | | | | | | 0 | | | | | | | 0.009 | | | | | | | 0.5 | | | | | | | 0.71 | | | | | | | | 0.2 | | | | 0.16 | | | |
| Site 58 | | | 0.92 | | | | | | | | | 0.26 | | | | | | | | | 0.07 | | | | | | | 0 | | | | | | | 0.3 | | | | | | | 0.82 | | | | | | | | 0.04 | | | | 0.18 | | | |
|  | | | | | | | | | | | | | | | | | | | | | | | | | | | | | | | | | | | | | | | | | | | | | | | | | | | | | | | | | |
| *A.saccharum* | | | | | | N | | | | | | | | VTX1 | | | | | | | | | VTX2 | | | | | | VTX3 | | | | | | | VTX4 | | | | | | | VTX7 | | | | | | | | VTX8 | | | | | | VTX9 |
| Site 6 | | | | | | 0.49 | | | | | | | | 0.07 | | | | | | | | | 0.32 | | | | | | 0.06 | | | | | | | 0.13 | | | | | | | 0.11 | | | | | | | | 0.24 | | | | | | 0.08 |
| Site 7 | | | | | | 1.04 | | | | | | | | 0.34 | | | | | | | | | 0.36 | | | | | | 0.41 | | | | | | | 0.45 | | | | | | | 0.11 | | | | | | | | 0.03 | | | | | | 0.06 |
| Site 22 | | | | | | 1.53 | | | | | | | | 0.31 | | | | | | | | | 0.48 | | | | | | 0.45 | | | | | | | 0.35 | | | | | | | 0.14 | | | | | | | | 0.15 | | | | | | 0.11 |
| Site 24 | | | | | | 1.22 | | | | | | | | 0.32 | | | | | | | | | 0.46 | | | | | | 0.47 | | | | | | | 0.22 | | | | | | | 0.08 | | | | | | | | 0.13 | | | | | | 0.07 |
| Site 41 | | | | | | 1.51 | | | | | | | | 0.33 | | | | | | | | | 0.54 | | | | | | 0.17 | | | | | | | 0.50 | | | | | | | 0.10 | | | | | | | | 0.13 | | | | | | 0.07 |
| Site 100 | | | | | | 1.35 | | | | | | | | 0.25 | | | | | | | | | 0.40 | | | | | | 0.13 | | | | | | | 0.43 | | | | | | | 0.14 | | | | | | | | 0.12 | | | | | | 0.10 |
|  | | | | | | | | | | | | | | | | | | | | | | | | | | | | | | | | | | | | | | | | | | | | | | | | | | | | | | | | | |
| *Q. rubra* | | | N | | | | | Russula | | | | | | | | Cortinarius | | | | | | | | | Piloderma | | | | | | | Inocybe | | | | | | Amanita | | | | | | | Tomentella | | | | | | | | Cenococcum | | | | |
| Site 3 | | | 0.65 | | | | | 0.25 | | | | | | | | 0.27 | | | | | | | | | 0.49 | | | | | | | 0.00 | | | | | | 0.21 | | | | | | | 0.06 | | | | | | | | 0.11 | | | | |
| Site 6 | | | 1.25 | | | | | 0.32 | | | | | | | | 0.09 | | | | | | | | | 0.08 | | | | | | | 0.16 | | | | | | 0.09 | | | | | | | 0.10 | | | | | | | | 0.02 | | | | |
| Site 7 | | | 0.88 | | | | | 0.46 | | | | | | | | 0.34 | | | | | | | | | 0.15 | | | | | | | 0.02 | | | | | | 0.06 | | | | | | | 0.13 | | | | | | | | 0.11 | | | | |
| Site 9 | | | 0.79 | | | | | 0.25 | | | | | | | | 0.13 | | | | | | | | | 0.05 | | | | | | | 0.05 | | | | | | 0.05 | | | | | | | 0.11 | | | | | | | | 0.10 | | | | |
| Site 20 | | | 0.64 | | | | | 0.26 | | | | | | | | 0.49 | | | | | | | | | 0.13 | | | | | | | 0.01 | | | | | | 0.07 | | | | | | | 0.08 | | | | | | | | 0.11 | | | | |
| Site 24 | | | 1.27 | | | | | 0.42 | | | | | | | | 0.28 | | | | | | | | | 0.06 | | | | | | | 0.25 | | | | | | 0.08 | | | | | | | 0.15 | | | | | | | | 0.11 | | | | |
| Site 22 | | | 1.46 | | | | | 0.38 | | | | | | | | 0.00 | | | | | | | | | 0.05 | | | | | | | 0.17 | | | | | | 0.02 | | | | | | | 0.10 | | | | | | | | 0.01 | | | | |
| Site 31 | | | 1.43 | | | | | 0.36 | | | | | | | | 0.32 | | | | | | | | | 0.21 | | | | | | | 0.04 | | | | | | 0.15 | | | | | | | 0.11 | | | | | | | | 0.15 | | | | |
| Site 41 | | | 1.53 | | | | | 0.39 | | | | | | | | 0.26 | | | | | | | | | 0.05 | | | | | | | 0.18 | | | | | | 0.11 | | | | | | | 0.16 | | | | | | | | 0.09 | | | | |
| Site 50 | | | 0.32 | | | | | 0.22 | | | | | | | | 0.33 | | | | | | | | | 0.49 | | | | | | | 0.00 | | | | | | 0.19 | | | | | | | 0.06 | | | | | | | | 0.11 | | | | |
| Site 58 | | | 1.08 | | | | | 0.26 | | | | | | | | 0.31 | | | | | | | | | 0.44 | | | | | | | 0.01 | | | | | | 0.14 | | | | | | | 0.06 | | | | | | | | 0.07 | | | | |
| Site 100 | | | 1.89 | | | | | 0.37 | | | | | | | | 0.17 | | | | | | | | | 0.12 | | | | | | | 0.07 | | | | | | 0.05 | | | | | | | 0.21 | | | | | | | | 0.05 | | | | |
| Mycorrhizal abundance (proportions) in low and high levels of the target taxa | | | | | | | | | | | | | | | | | | | | | | | | | | | | | | | | | | | | | | | | | | | | | | | | | | | | | | | | | |
| *A.rubrum* | | | | | | | | | | | | | | | | | | | | | | | | | | | | | | | | | | | | | | | | | | | | | | | | | | | | | | | | | |
| Target taxa | | | | level | | | | | VTX1 | | | | | | | | | | VTX2 | | | | | | | | VTX3 | | | | | | | VTX4 | | | | | | VTX5 | | | | | | | VTX6 | | | | | | | VTX7 | | | |
| VTX1 | | | | low | | | | | 0.39 | | | | | | | | | | 0.03 | | | | | | | | 0.00 | | | | | | | 0.19 | | | | | | 0.87 | | | | | | | 0.10 | | | | | | | 0.00 | | | |
| VTX2 | | | | low | | | | | 0.07 | | | | | | | | | | 0.00 | | | | | | | | 0.00 | | | | | | | 0.33 | | | | | | 0.86 | | | | | | | 0.19 | | | | | | | 0.14 | | | |
| VTX3 | | | | low | | | | | 0.06 | | | | | | | | | | 0.00 | | | | | | | | 0.01 | | | | | | | 0.46 | | | | | | 0.75 | | | | | | | 0.19 | | | | | | | 0.19 | | | |
| VTX4 | | | | low | | | | | 0.06 | | | | | | | | | | 0.00 | | | | | | | | 0.01 | | | | | | | 0.46 | | | | | | 0.75 | | | | | | | 0.19 | | | | | | | 0.19 | | | |
| VTX5 | | | | low | | | | | 0.52 | | | | | | | | | | 0.39 | | | | | | | | 0.16 | | | | | | | 0.37 | | | | | | 0.21 | | | | | | | 0.00 | | | | | | | 0.07 | | | |
| VTX6 | | | | low | | | | | 0.46 | | | | | | | | | | 0.27 | | | | | | | | 0.10 | | | | | | | 0.09 | | | | | | 0.58 | | | | | | | 0.03 | | | | | | | 0.04 | | | |
| VTX7 | | | | low | | | | | 0.37 | | | | | | | | | | 0.22 | | | | | | | | 0.08 | | | | | | | 0.11 | | | | | | 0.63 | | | | | | | 0.13 | | | | | | | 0.07 | | | |
| VTX1 | | | | high | | | | | 0.60 | | | | | | | | | | 0.40 | | | | | | | | 0.12 | | | | | | | 0.39 | | | | | | 0.34 | | | | | | | 0.02 | | | | | | | 0.08 | | | |
| VTX2 | | | | high | | | | | 0.45 | | | | | | | | | | 0.54 | | | | | | | | 0.15 | | | | | | | 0.30 | | | | | | 0.37 | | | | | | | 0.03 | | | | | | | 0.09 | | | |
| VTX3 | | | | high | | | | | 0.38 | | | | | | | | | | 0.37 | | | | | | | | 0.42 | | | | | | | 0.38 | | | | | | 0.30 | | | | | | | 0.06 | | | | | | | 0.08 | | | |
| VTX4 | | | | high | | | | | 0.13 | | | | | | | | | | 0.12 | | | | | | | | 0.12 | | | | | | | 0.46 | | | | | | 0.67 | | | | | | | 0.12 | | | | | | | 0.23 | | | |
| VTX5 | | | | high | | | | | 0.22 | | | | | | | | | | 0.02 | | | | | | | | 0.00 | | | | | | | 0.26 | | | | | | 0.90 | | | | | | | 0.05 | | | | | | | 0.09 | | | |
| VTX6 | | | | high | | | | | 0.25 | | | | | | | | | | 0.04 | | | | | | | | 0.00 | | | | | | | 0.30 | | | | | | 0.81 | | | | | | | 0.30 | | | | | | | 0.07 | | | |
| VTX7 | | | | high | | | | | 0.38 | | | | | | | | | | 0.17 | | | | | | | | 0.02 | | | | | | | 0.74 | | | | | | 0.37 | | | | | | | 0.01 | | | | | | | 0.10 | | | |
|  | | | | | | | | | | | | | | | | | | | | | | | | | | | | | | | | | | | | | | | | | | | | | | | | | | | | | | | | | |
| *A.saccharum* | | | | | | | | | | | | | | | | | | | | | | | | | | | | | | | | | | | | | | | | | | | | | | | | | | | | | | | | | |
| Target taxa | level | | | | | | VTX1 | | | | | | | | | | VTX2 | | | | | | | | | VTX3 | | | | | | | VTX4 | | | | | | | | VTX7 | | | | | | | | VTX8 | | | | | | | VTX9 | |
| VTX1 | low | | | | | | 0.05 | | | | | | | | | | 0.28 | | | | | | | | | 0.05 | | | | | | | 0.12 | | | | | | | | 0.13 | | | | | | | | 0.20 | | | | | | | 0.10 | |
| VTX2 | low | | | | | | 0.20 | | | | | | | | | | 0.23 | | | | | | | | | 0.20 | | | | | | | 0.21 | | | | | | | | 0.12 | | | | | | | | 0.12 | | | | | | | 0.09 | |
| VTX3 | low | | | | | | 0.08 | | | | | | | | | | 0.31 | | | | | | | | | 0.01 | | | | | | | 0.24 | | | | | | | | 0.13 | | | | | | | | 0.19 | | | | | | | 0.10 | |
| VTX4 | low | | | | | | 0.18 | | | | | | | | | | 0.37 | | | | | | | | | 0.23 | | | | | | | 0.08 | | | | | | | | 0.12 | | | | | | | | 0.18 | | | | | | | 0.10 | |
| VTX7 | low | | | | | | 0.29 | | | | | | | | | | 0.38 | | | | | | | | | 0.33 | | | | | | | 0.35 | | | | | | | | 0.06 | | | | | | | | 0.11 | | | | | | | 0.05 | |
| VTX8 | low | | | | | | 0.33 | | | | | | | | | | 0.40 | | | | | | | | | 0.38 | | | | | | | 0.40 | | | | | | | | 0.10 | | | | | | | | 0.02 | | | | | | | 0.06 | |
| VTX9 | low | | | | | | 0.30 | | | | | | | | | | 0.36 | | | | | | | | | 0.34 | | | | | | | 0.41 | | | | | | | | 0.07 | | | | | | | | 0.10 | | | | | | | 0.04 | |
| VTX1 | high | | | | | | 0.45 | | | | | | | | | | 0.42 | | | | | | | | | 0.36 | | | | | | | 0.35 | | | | | | | | 0.10 | | | | | | | | 0.11 | | | | | | | 0.08 | |
| VTX2 | high | | | | | | 0.31 | | | | | | | | | | 0.61 | | | | | | | | | 0.28 | | | | | | | 0.43 | | | | | | | | 0.14 | | | | | | | | 0.16 | | | | | | | 0.09 | |
| VTX3 | high | | | | | | 0.33 | | | | | | | | | | 0.33 | | | | | | | | | 0.53 | | | | | | | 0.30 | | | | | | | | 0.10 | | | | | | | | 0.11 | | | | | | | 0.07 | |
| VTX4 | high | | | | | | 0.28 | | | | | | | | | | 0.48 | | | | | | | | | 0.22 | | | | | | | 0.62 | | | | | | | | 0.09 | | | | | | | | 0.10 | | | | | | | 0.06 | |
| VTX7 | high | | | | | | 0.25 | | | | | | | | | | 0.45 | | | | | | | | | 0.28 | | | | | | | 0.34 | | | | | | | | 0.17 | | | | | | | | 0.11 | | | | | | | 0.11 | |
| VTX8 | high | | | | | | 0.11 | | | | | | | | | | 0.39 | | | | | | | | | 0.14 | | | | | | | 0.13 | | | | | | | | 0.10 | | | | | | | | 0.24 | | | | | | | 0.08 | |
| VTX9 | high | | | | | | 0.18 | | | | | | | | | | 0.39 | | | | | | | | | 0.26 | | | | | | | 0.29 | | | | | | | | 0.16 | | | | | | | | 0.16 | | | | | | | 0.12 | |
|  | | | | | | | | | | | | | | | | | | | | | | | | | | | | | | | | | | | | | | | | | | | | | | | | | | | | | | | | | |
| *Q. rubra* | | | | | | | | | | | | | | | | | | | | | | | | | | | | | | | | | | | | | | | | | | | | | | | | | | | | | | | | | |
| Target taxa | | | | | level | | | | | Russula | | | | | | | | Cortinarius | | | | | | | | | Piloderma | | | | | | | Inocybe | | | | | | Amanita | | | | | | | Tomentella | | | | | | | Cenococcum | | | |
| Russula | | | | | low | | | | | 0.58 | | | | | | | | 0.29 | | | | | | | | | 0.12 | | | | | | | 0.04 | | | | | | 0.10 | | | | | | | 0.12 | | | | | | | 0.08 | | | |
| Cortinarius | | | | | low | | | | | 0.25 | | | | | | | | 0.59 | | | | | | | | | 0.15 | | | | | | | 0.11 | | | | | | 0.09 | | | | | | | 0.09 | | | | | | | 0.11 | | | |
| Piloderma | | | | | low | | | | | 0.21 | | | | | | | | 0.20 | | | | | | | | | 0.66 | | | | | | | 0.00 | | | | | | 0.25 | | | | | | | 0.05 | | | | | | | 0.08 | | | |
| Inocybe | | | | | low | | | | | 0.31 | | | | | | | | 0.25 | | | | | | | | | 0.05 | | | | | | | 0.43 | | | | | | 0.04 | | | | | | | 0.12 | | | | | | | 0.08 | | | |
| Amanita | | | | | low | | | | | 0.35 | | | | | | | | 0.17 | | | | | | | | | 0.46 | | | | | | | 0.05 | | | | | | 0.35 | | | | | | | 0.06 | | | | | | | 0.10 | | | |
| Tomentella | | | | | low | | | | | 0.46 | | | | | | | | 0.12 | | | | | | | | | 0.11 | | | | | | | 0.19 | | | | | | 0.05 | | | | | | | 0.31 | | | | | | | 0.07 | | | |
| Cenococcum | | | | | low | | | | | 0.21 | | | | | | | | 0.34 | | | | | | | | | 0.26 | | | | | | | 0.00 | | | | | | 0.13 | | | | | | | 0.08 | | | | | | | 0.20 | | | |
| Russula | | | | | high | | | | | 0.10 | | | | | | | | 0.22 | | | | | | | | | 0.22 | | | | | | | 0.09 | | | | | | 0.07 | | | | | | | 0.10 | | | | | | | 0.07 | | | |
| Cortinarius | | | | | high | | | | | 0.35 | | | | | | | | 0.00 | | | | | | | | | 0.05 | | | | | | | 0.15 | | | | | | 0.02 | | | | | | | 0.09 | | | | | | | 0.01 | | | |
| Piloderma | | | | | high | | | | | 0.29 | | | | | | | | 0.09 | | | | | | | | | 0.00 | | | | | | | 0.15 | | | | | | 0.05 | | | | | | | 0.06 | | | | | | | 0.05 | | | |
| Inocybe | | | | | high | | | | | 0.29 | | | | | | | | 0.16 | | | | | | | | | 0.01 | | | | | | | 0.00 | | | | | | 0.08 | | | | | | | 0.07 | | | | | | | 0.09 | | | |
| Amanita | | | | | high | | | | | 0.46 | | | | | | | | 0.17 | | | | | | | | | 0.08 | | | | | | | 0.05 | | | | | | 0.00 | | | | | | | 0.14 | | | | | | | 0.04 | | | |
| Tomentella | | | | | high | | | | | 0.31 | | | | | | | | 0.09 | | | | | | | | | 0.15 | | | | | | | 0.04 | | | | | | 0.07 | | | | | | | 0.02 | | | | | | | 0.04 | | | |
| Cenococcum | | | | | high | | | | | 0.34 | | | | | | | | 0.01 | | | | | | | | | 0.05 | | | | | | | 0.09 | | | | | | 0.03 | | | | | | | 0.05 | | | | | | | 0.00 | | | |

**Table S5:** Parameter values from the analyses, means, SDs and 95% CIs. Values in figures in the main text have been standardized by multiplied these parameter values by the average value of the covariate. Bold values indicate that covariate is significantly associated with tree growth (95%CI does not overlap with zero).

| *Acer rubrum* | mean | | SD | 2.5% | 97.5% |
| --- | --- | --- | --- | --- | --- |
| Intercept α_o_ | -6.59 | | 1.12 | -8.63 | -4.35 |
| ln(dbh) α_1_ | **1.38** | | **0.04** | **1.29** | **1.46** |
| N miner α_2_ | **3.58** | | **1.20** | **1.10** | **5.85** |
| BAIS α_3_ | **0.20** | | **0.01** | **0.18** | **0.22** |
| age α_4_ | **-0.01** | | **0.00** | **-0.01** | **-0.01** |
| minMayTemp α_5_ | **0.03** | | **0.01** | **0.02** | **0.04** |
| AMF neigh α_6_ | **0.01** | | **0.00** | **0.01** | **0.02** |
| EMF neigh α_7_ | **-0.01** | | **0.00** | **-0.01** | **-0.01** |
| VTX1 β_1_ | -0.37 | | 0.53 | -1.39 | 0.63 |
| VTX2 β_2_ | **1.82** | | **0.62** | **0.46** | **2.90** |
| VTX3 β_3_ | **1.92** | | **0.67** | **0.63** | **3.31** |
| VTX4 β_4_ | **-2.27** | | **0.44** | **-3.14** | **-1.43** |
| VTX5 β_5_ | **7.05** | | **1.32** | **4.39** | **9.34** |
| VTX6 β_6_ | **-9.65** | | **0.96** | **-11.23** | **-7.53** |
| VTX7 β_7_ | **-4.43** | | **1.12** | **-6.61** | **-2.11** |
| VTX1^2^ γ_1_ | 0.29 | | 0.84 | -1.47 | 1.70 |
| VTX2^2^ γ_2_ | **2.15** | | **0.84** | **0.36** | **3.92** |
| VTX3^2^ γ_3_ | **14.06** | | **1.56** | **10.10** | **16.40** |
| VTX4^2^ γ_4_ | **6.28** | | **0.83** | **4.72** | **7.85** |
| VTX5^2^ γ_5_ | **-0.96** | | **0.61** | **-2.43** | **-0.03** |
| VTX6^2^ γ_6_ | **11.65** | | **1.50** | **8.21** | **14.13** |
| VTX7^2^ γ_7_ | **-4.10** | | **1.90** | **-7.86** | **-0.43** |
| N min⋅VTX1 μ_1_ | 0.44 | | 0.52 | -0.53 | 1.53 |
| N min⋅VTX2 μ_2_ | **-2.45** | | **0.79** | **-3.99** | **-0.87** |
| N min⋅VTX3 μ_3_ | **-5.36** | | **0.74** | **-6.69** | **-3.90** |
| N min⋅VTX4 μ_4_ | **-1.93** | | **0.77** | **-3.22** | **-0.40** |
| N min⋅VTX5 μ_5_ | **-4.88** | | **1.10** | **-6.79** | **-2.43** |
| N min⋅VTX6 μ_6_ | **7.05** | | **0.77** | **5.40** | **8.39** |
| N min⋅VTX7 μ_7_ | **5.87** | | **1.50** | **2.34** | **8.37** |
| var intercept *a* | 0.29 | | 0.06 | 0.17 | 0.40 |
| var ln(dbh) *b* | -0.04 | | 0.02 | -0.07 | 0.00 |
|  | | | | | |
| *Acer saccharum* | mean | | SD | 2.5% | 97.5% |
| Intercept α_o_ | -4.35 | | 0.41 | -5.25 | -3.68 |
| ln(dbh) α_1_ | **1.49** | | **0.08** | **1.35** | **1.65** |
| N miner α_2_ | **1.10** | | **0.39** | **0.07** | **1.66** |
| BAIS α_3_ | **0.17** | | **0.01** | **0.15** | **0.19** |
| age α_4_ | **-0.01** | | **0.00** | **-0.01** | **-0.01** |
| VTX1 β_1_ | 0.00 | | 0.01 | -0.01 | 0.01 |
| VTX2 β_2_ | **1.34** | | **0.65** | **0.14** | **2.57** |
| VTX3 β_3_ | **2.67** | | **0.73** | **1.34** | **3.96** |
| VTX4 β_4_ | 0.54 | | 0.46 | -0.30 | 1.44 |
| VTX7 β_5_ | **-1.03** | | **0.46** | **-1.96** | **-0.16** |
| VTX8 β_6_ | **2.57** | | **1.98** | **0.59** | **7.09** |
| VTX9 β_7_ | 0.07 | | 1.05 | -1.85 | 1.99 |
| VTX1^2^ γ_1_ | 2.73 | | 1.95 | -2.15 | 5.84 |
| VTX2^2^ γ_2_ | **3.74** | | **0.94** | **2.03** | **5.56** |
| VTX3^2^ γ_3_ | **-2.43** | | **1.08** | **-4.82** | **-0.49** |
| VTX4^2^ γ_4_ | 0.15 | | 0.76 | -1.08 | 1.84 |
| VTX7^2^ γ_5_ | -1.10 | | 0.62 | -2.20 | 0.04 |
| VTX8^2^ γ_6_ | -1.06 | | 2.35 | -5.69 | 4.32 |
| VTX9^2^ γ_7_ | 4.79 | | 3.06 | -0.03 | 11.86 |
| N min⋅VTX1 μ_1_ | 0.74 | | 2.23 | -4.20 | 4.54 |
| N min⋅VTX2 μ_2_ | **-3.10** | | **0.45** | **-3.95** | **-2.20** |
| N min⋅VTX3 μ_3_ | -1.07 | | 0.79 | -2.29 | 0.88 |
| N min⋅VTX4 μ_4_ | -0.14 | | 0.26 | -0.63 | 0.38 |
| N min⋅VTX7 μ_5_ | 1.67 | | 0.26 | 1.20 | 2.23 |
| N min⋅VTX8 μ_6_ | 1.64 | | 1.43 | -1.66 | 3.51 |
| N min⋅VTX9 μ_7_ | **1.56** | | **0.82** | **0.22** | **3.38** |
| var intercept *a* | -7.66 | | 1.79 | -10.89 | -3.92 |
| var ln(dbh) *b* | 0.00 | | 0.01 | 0.00 | 0.05 |
|  | | | | | |
| *Quercus rubra* | | mean | SD | 2.5% | 97.5% |
| Intercept α_o_ | | -0.97 | 0.11 | -1.20 | -0.76 |
| ln(dbh) α_1_ | | **1.22** | **0.03** | **1.17** | **1.27** |
| N miner α_2_ | | **-0.71** | **0.07** | **-0.85** | **-0.58** |
| BAIS α_3_ | | **0.13** | **0.01** | **0.12** | **0.14** |
| age α_4_ | | **-0.01** | **0.00** | **-0.01** | **-0.01** |
| Russula β_1_ | | **0.01** | **0.00** | **0.01** | **0.02** |
| Continarius β_2_ | | -0.30 | 0.20 | -0.68 | 0.09 |
| Piloderma β_3_ | | **-0.53** | **0.24** | **-1.08** | **-0.14** |
| Inocybe β_4_ | | **-0.97** | **0.17** | **-1.33** | **-0.63** |
| Amanita β_5_ | | -0.07 | 0.20 | -0.49 | 0.31 |
| Tomentella β_6_ | | 0.06 | 0.32 | -0.53 | 0.71 |
| Cenococcum β_7_ | | **1.28** | **0.48** | **0.47** | **2.22** |
| Russula ^2^ γ_1_ | | **-1.17** | **0.64** | **-2.80** | **-0.40** |
| Continarius ^2^ γ_2_ | | 0.21 | 0.33 | -0.45 | 0.82 |
| Piloderma ^2^ γ_3_ | | -0.41 | 0.30 | -0.94 | 0.22 |
| Inocybe ^2^ γ_4_ | | **0.43** | **0.20** | **0.05** | **0.82** |
| Amanita^2^ γ_5_ | | **-1.03** | **0.46** | **-1.94** | **-0.13** |
| Tomentella ^2^ γ_6_ | | **1.28** | **0.42** | **0.47** | **2.13** |
| Cenococcum ^2^ γ_7_ | | **-18.08** | **1.11** | **-19.98** | **-15.73** |
| N min⋅ Russula μ_1_ | | **4.79** | **1.43** | **2.42** | **7.77** |
| N min⋅ Continarius μ_2_ | | **0.45** | **0.13** | **0.20** | **0.69** |
| N min⋅ Piloderma μ_3_ | | **0.30** | **0.10** | **0.13** | **0.52** |
| N min⋅ Inocybe μ_4_ | | **0.37** | **0.11** | **0.16** | **0.59** |
| N min⋅ Amanita μ_5_ | | **0.43** | **0.15** | **0.15** | **0.73** |
| N min⋅ Tomentella μ_6_ | | -0.18 | 0.24 | -0.66 | 0.30 |
| N min⋅ Cenococcum μ_7_ | | **2.96** | **0.25** | **2.49** | **3.44** |
| var intercept *a* | | -0.03 | 0.36 | -0.68 | 0.71 |
| var ln(dbh) *b* | | 0.12 | 0.06 | 0.00 | 0.17 |

**Table S6:** Correlations between parameters associated with mycorrhizal abundance. Bold indicates correlation coefficient is significantly different from zero.

| *Acer rubrum* | | | | | |
| --- | --- | --- | --- | --- | --- |
| Parameter | Taxon 1 | Taxon 2 | mean | 2.5% | 97.5% |
| β | VTX1 | VTX1 | 1.00 | 1.00 | 1.00 |
| β | VTX1 | VTX2 | 0.00 | -0.67 | 0.67 |
| β | VTX1 | VTX3 | 0.00 | -0.66 | 0.67 |
| β | VTX1 | VTX4 | -0.01 | -0.67 | 0.66 |
| β | VTX1 | VTX5 | 0.02 | -0.66 | 0.68 |
| β | VTX1 | VTX6 | -0.04 | -0.69 | 0.65 |
| β | VTX1 | VTX7 | -0.01 | -0.67 | 0.66 |
| β | VTX2 | VTX2 | 1.00 | 1.00 | 1.00 |
| β | VTX2 | VTX3 | -0.02 | -0.68 | 0.65 |
| β | VTX2 | VTX4 | 0.03 | -0.65 | 0.68 |
| β | VTX2 | VTX5 | -0.09 | -0.71 | 0.62 |
| β | VTX2 | VTX6 | 0.15 | -0.58 | 0.75 |
| β | VTX2 | VTX7 | 0.05 | -0.64 | 0.69 |
| β | VTX3 | VTX3 | 1.00 | 1.00 | 1.00 |
| β | VTX3 | VTX4 | 0.03 | -0.65 | 0.68 |
| β | VTX3 | VTX5 | -0.10 | -0.72 | 0.61 |
| β | VTX3 | VTX6 | 0.17 | -0.57 | 0.75 |
| β | VTX3 | VTX7 | 0.05 | -0.63 | 0.70 |
| β | VTX4 | VTX4 | 1.00 | 1.00 | 1.00 |
| β | VTX4 | VTX5 | 0.11 | -0.60 | 0.73 |
| β | VTX4 | VTX6 | -0.20 | -0.77 | 0.54 |
| β | VTX4 | VTX7 | -0.07 | -0.70 | 0.63 |
| β | VTX5 | VTX5 | 1.00 | 1.00 | 1.00 |
| **β** | **VTX5** | **VTX6** | **0.68** | **0.00** | **0.93** |
| β | VTX5 | VTX7 | 0.24 | -0.52 | 0.78 |
| β | VTX6 | VTX6 | 1.00 | 1.00 | 1.00 |
| β | VTX6 | VTX7 | -0.40 | -0.85 | 0.37 |
| β | VTX7 | VTX7 | 1.00 | 1.00 | 1.00 |
| γ | VTX1 | VTX1 | 1.00 | 1.00 | 1.00 |
| γ | VTX1 | VTX2 | 0.00 | -0.67 | 0.66 |
| γ | VTX1 | VTX3 | -0.02 | -0.68 | 0.66 |
| γ | VTX1 | VTX4 | 0.00 | -0.67 | 0.67 |
| γ | VTX1 | VTX5 | 0.00 | -0.67 | 0.67 |
| γ | VTX1 | VTX6 | -0.01 | -0.67 | 0.66 |
| γ | VTX1 | VTX7 | 0.00 | -0.66 | 0.67 |
| γ | VTX2 | VTX2 | 1.00 | 1.00 | 1.00 |
| γ | VTX2 | VTX3 | -0.12 | -0.73 | 0.60 |
| γ | VTX2 | VTX4 | -0.04 | -0.69 | 0.64 |
| γ | VTX2 | VTX5 | 0.01 | -0.66 | 0.67 |
| γ | VTX2 | VTX6 | -0.08 | -0.71 | 0.62 |
| γ | VTX2 | VTX7 | 0.02 | -0.65 | 0.68 |
| γ | VTX3 | VTX3 | 1.00 | 1.00 | 1.00 |
| γ | VTX3 | VTX4 | -0.35 | -0.82 | 0.42 |
| γ | VTX3 | VTX5 | 0.05 | -0.64 | 0.69 |
| **γ** | **VTX3** | **VTX6** | **-0.73** | **-0.94** | **-0.11** |
| γ | VTX3 | VTX7 | 0.22 | -0.54 | 0.78 |
| γ | VTX4 | VTX4 | 1.00 | 1.00 | 1.00 |
| γ | VTX4 | VTX5 | 0.02 | -0.66 | 0.68 |
| γ | VTX4 | VTX6 | -0.26 | -0.79 | 0.49 |
| γ | VTX4 | VTX7 | 0.08 | -0.62 | 0.71 |
| γ | VTX5 | VTX5 | 1.00 | 1.00 | 1.00 |
| γ | VTX5 | VTX6 | 0.04 | -0.64 | 0.69 |
| γ | VTX5 | VTX7 | -0.01 | -0.67 | 0.66 |
| γ | VTX6 | VTX6 | 1.00 | 1.00 | 1.00 |
| γ | VTX6 | VTX7 | 0.17 | -0.57 | 0.75 |
| γ | VTX7 | VTX7 | 1.00 | 1.00 | 1.00 |
| *Acer saccharum* | | | | | |
| Parameter | Taxon 1 | Taxon 2 | mean | 2.5% | 97.5% |
| β | VTX1 | VTX1 | 1.00 | 1.00 | 1.00 |
| β | VTX1 | VTX2 | -0.13 | -0.74 | 0.59 |
| β | VTX1 | VTX3 | -0.07 | -0.71 | 0.63 |
| β | VTX1 | VTX4 | 0.04 | -0.64 | 0.69 |
| β | VTX1 | VTX7 | -0.21 | -0.80 | 0.56 |
| β | VTX1 | VTX8 | -0.06 | -0.72 | 0.64 |
| β | VTX1 | VTX9 | -0.20 | -0.79 | 0.58 |
| β | VTX2 | VTX2 | 1.00 | 1.00 | 1.00 |
| β | VTX2 | VTX3 | -0.07 | -0.71 | 0.63 |
| β | VTX2 | VTX4 | 0.07 | -0.63 | 0.71 |
| β | VTX2 | VTX7 | -0.29 | -0.86 | 0.54 |
| β | VTX2 | VTX8 | -0.05 | -0.70 | 0.64 |
| β | VTX2 | VTX9 | -0.28 | -0.84 | 0.54 |
| β | VTX3 | VTX3 | 1.00 | 1.00 | 1.00 |
| β | VTX3 | VTX4 | 0.03 | -0.65 | 0.68 |
| β | VTX3 | VTX7 | -0.10 | -0.73 | 0.62 |
| β | VTX3 | VTX8 | -0.04 | -0.70 | 0.65 |
| β | VTX3 | VTX9 | -0.11 | -0.74 | 0.61 |
| β | VTX4 | VTX4 | 1.00 | 1.00 | 1.00 |
| β | VTX4 | VTX7 | 0.11 | -0.62 | 0.74 |
| β | VTX4 | VTX8 | 0.02 | -0.65 | 0.68 |
| β | VTX4 | VTX9 | 0.11 | -0.62 | 0.75 |
| **β** | VTX7 | VTX7 | 1.00 | 1.00 | 1.00 |
| β | VTX7 | VTX8 | -0.09 | -0.74 | 0.63 |
| β | VTX7 | VTX9 | -0.28 | -0.83 | 0.59 |
| β | VTX8 | VTX8 | 1.00 | 1.00 | 1.00 |
| β | VTX8 | VTX9 | -0.07 | -0.72 | 0.66 |
| β | VTX9 | VTX9 | 1.00 | 1.00 | 1.00 |
| γ | VTX1 | VTX1 | 1.00 | 1.00 | 1.00 |
| γ | VTX1 | VTX2 | 0.34 | -0.51 | 0.89 |
| γ | VTX1 | VTX3 | 0.03 | -0.66 | 0.70 |
| γ | VTX1 | VTX4 | 0.14 | -0.60 | 0.76 |
| γ | VTX1 | VTX7 | -0.12 | -0.79 | 0.67 |
| γ | VTX1 | VTX8 | -0.35 | -0.86 | 0.49 |
| γ | VTX1 | VTX9 | -0.03 | -0.72 | 0.69 |
| γ | VTX2 | VTX2 | 1.00 | 1.00 | 1.00 |
| γ | VTX2 | VTX3 | -0.03 | -0.69 | 0.65 |
| γ | VTX2 | VTX4 | -0.07 | -0.71 | 0.63 |
| γ | VTX2 | VTX7 | 0.09 | -0.64 | 0.74 |
| γ | VTX2 | VTX8 | 0.20 | -0.56 | 0.79 |
| γ | VTX2 | VTX9 | 0.02 | -0.68 | 0.69 |
| γ | VTX3 | VTX3 | 1.00 | 1.00 | 1.00 |
| **γ** | VTX3 | VTX4 | -0.02 | -0.68 | 0.66 |
| γ | VTX3 | VTX7 | 0.02 | -0.66 | 0.69 |
| γ | VTX3 | VTX8 | 0.02 | -0.66 | 0.69 |
| γ | VTX3 | VTX9 | -0.01 | -0.67 | 0.67 |
| γ | VTX4 | VTX4 | 1.00 | 1.00 | 1.00 |
| γ | VTX4 | VTX7 | 0.04 | -0.65 | 0.71 |
| γ | VTX4 | VTX8 | 0.09 | -0.62 | 0.72 |
| γ | VTX4 | VTX9 | 0.00 | -0.67 | 0.67 |
| γ | VTX7 | VTX7 | 1.00 | 1.00 | 1.00 |
| γ | VTX7 | VTX8 | -0.06 | -0.78 | 0.75 |
| γ | VTX7 | VTX9 | -0.01 | -0.69 | 0.68 |
| γ | VTX8 | VTX8 | 1.00 | 1.00 | 1.00 |
| γ | VTX8 | VTX9 | -0.06 | -0.76 | 0.67 |
| γ | VTX9 | VTX9 | 1.00 | 1.00 | 1.00 |
| *Quercus rubra* | | | | | |
| Parameter | Taxon 1 | Taxon 2 | mean | 2.5% | 97.5% |
| β | Russula | Russula | 1.00 | 1.00 | 1.00 |
| β | Russula | Cortinarius | -0.03 | -0.69 | 0.65 |
| β | Russula | Piloderma | -0.06 | -0.71 | 0.63 |
| β | Russula | Inocybe | 0.00 | -0.67 | 0.66 |
| β | Russula | Amanita | 0.00 | -0.67 | 0.67 |
| β | Russula | Tomentella | 0.08 | -0.63 | 0.71 |
| β | Russula | Cenococcum | -0.07 | -0.71 | 0.62 |
| β | Cortinarius | Cortinarius | 1.00 | 1.00 | 1.00 |
| β | Cortinarius | Piloderma | -0.12 | -0.73 | 0.60 |
| β | Cortinarius | Inocybe | 0.00 | -0.67 | 0.67 |
| β | Cortinarius | Amanita | 0.00 | -0.67 | 0.67 |
| β | Cortinarius | Tomentella | 0.16 | -0.58 | 0.75 |
| β | Cortinarius | Cenococcum | -0.14 | -0.75 | 0.58 |
| β | Piloderma | Piloderma | 1.00 | 1.00 | 1.00 |
| β | Piloderma | Inocybe | -0.01 | -0.67 | 0.66 |
| β | Piloderma | Amanita | 0.01 | -0.67 | 0.68 |
| β | Piloderma | Tomentella | 0.28 | -0.49 | 0.81 |
| β | Piloderma | Cenococcum | -0.27 | -0.80 | 0.50 |
| β | Inocybe | Inocybe | 1.00 | 1.00 | 1.00 |
| β | Inocybe | Amanita | 0.00 | -0.67 | 0.67 |
| β | Inocybe | Tomentella | 0.01 | -0.66 | 0.68 |
| β | Inocybe | Cenococcum | -0.02 | -0.68 | 0.65 |
| **β** | Amanita | Amanita | 1.00 | 1.00 | 1.00 |
| β | Amanita | Tomentella | -0.02 | -0.68 | 0.66 |
| β | Amanita | Cenococcum | 0.01 | -0.67 | 0.69 |
| β | Tomentella | Tomentella | 1.00 | 1.00 | 1.00 |
| β | Tomentella | Cenococcum | 0.40 | -0.43 | 0.88 |
| β | Cenococcum | Cenococcum | 1.00 | 1.00 | 1.00 |
| γ | Russula | Russula | 1.00 | 1.00 | 1.00 |
| γ | Russula | Cortinarius | 0.00 | -0.67 | 0.66 |
| γ | Russula | Piloderma | 0.00 | -0.67 | 0.66 |
| γ | Russula | Inocybe | 0.00 | -0.67 | 0.67 |
| γ | Russula | Amanita | 0.00 | -0.67 | 0.67 |
| γ | Russula | Tomentella | 0.05 | -0.65 | 0.69 |
| γ | Russula | Cenococcum | 0.00 | -0.67 | 0.66 |
| γ | Cortinarius | Cortinarius | 1.00 | 1.00 | 1.00 |
| γ | Cortinarius | Piloderma | 0.00 | -0.67 | 0.67 |
| γ | Cortinarius | Inocybe | 0.00 | -0.67 | 0.67 |
| γ | Cortinarius | Amanita | 0.00 | -0.67 | 0.67 |
| γ | Cortinarius | Tomentella | -0.08 | -0.71 | 0.62 |
| γ | Cortinarius | Cenococcum | 0.01 | -0.66 | 0.67 |
| γ | Piloderma | Piloderma | 1.00 | 1.00 | 1.00 |
| **γ** | Piloderma | Inocybe | 0.00 | -0.67 | 0.67 |
| γ | Piloderma | Amanita | 0.00 | -0.67 | 0.67 |
| γ | Piloderma | Tomentella | 0.09 | -0.62 | 0.71 |
| γ | Piloderma | Cenococcum | -0.01 | -0.67 | 0.66 |
| γ | Inocybe | Inocybe | 1.00 | 1.00 | 1.00 |
| γ | Inocybe | Amanita | 0.00 | -0.66 | 0.67 |
| γ | Inocybe | Tomentella | -0.21 | -0.78 | 0.55 |
| γ | Inocybe | Cenococcum | 0.01 | -0.66 | 0.68 |
| γ | Amanita | Amanita | 1.00 | 1.00 | 1.00 |
| γ | Amanita | Tomentella | 0.26 | -0.51 | 0.80 |
| γ | Amanita | Cenococcum | -0.02 | -0.68 | 0.65 |
| γ | Tomentella | Tomentella | 1.00 | 1.00 | 1.00 |
| γ | **Tomentella** | **Cenococcum** | **0.92** | **0.55** | **0.99** |
| γ | Cenococcum | Cenococcum | 1.00 | 1.00 | 1.00 |

Supplementary Figures

**Figure S1**: Location of our study region in the USA (left) and of the 12 sampled locations (right; red circles) that span a N mineralization gradient in Michigan Lower Peninsula, USA (site ID numbers [Table S1] inside circles). QGIS.org (2023). QGIS Geographic Information System. Open Source Geospatial Foundation Project. <http://qgis.org>. More location details in Table S1.

**Figure S2**: Predicted vs observed tree growth (BAI) graphs, i.e., goodness of fit, for the three tree species analyzed. Correlations of residuals with phosphorous were: 0.01 for *A. rubrum*, 0.01 for *A. saccharum* and 0.1 for *Q. rubra*.


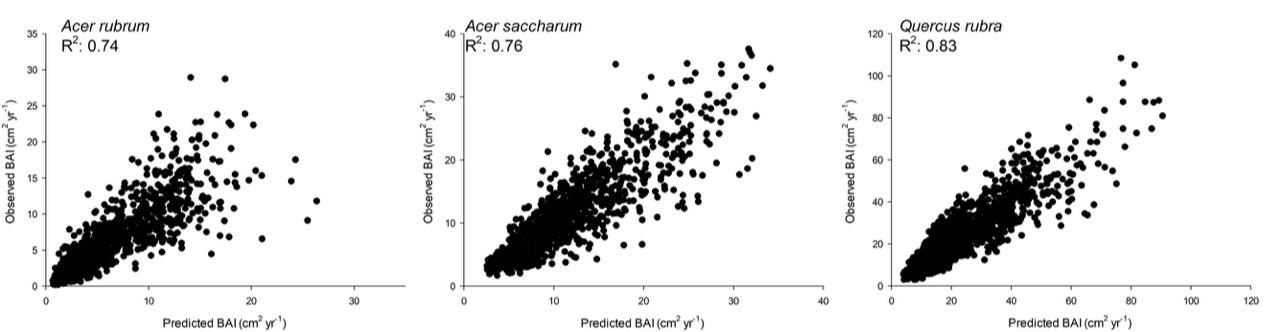


**Figure S3:** To show the previously described relationship between Chao1 AMF species richness and the (a) mean BAI per tree over the last 41 years representative of *Acer rubrum* and *A. saccharum* growth and (b) the net N mineralization gradient. The plots represent significant regressions with Chao1 species richness as the dependent variable and (a) mean BAI and (b) N mineralization gradient as the independent variables, from which the adjusted R^2^ was calculated, the shaded area represents 95% confidence intervals.
